# Dysregulation of MITF Leads to Transformation in MC1R-Defective Melanocytes

**DOI:** 10.3390/cancers12071719

**Published:** 2020-06-28

**Authors:** Timothy J. Lavelle, Tine Norman Alver, Karen-Marie Heintz, Patrik Wernhoff, Vegard Nygaard, Sigve Nakken, Geir Frode Øy, Sigurd Leinæs Bøe, Alfonso Urbanucci, Eivind Hovig

**Affiliations:** 1 Department of Tumor Biology, Institute for Cancer Research, Oslo University Hospital, 0424 Oslo, Norway; timothy.j.lavelle@rr-research.no (T.J.L.); tine.norman.alver@rr-research.no (T.N.A.); Karen-Marie.Heintz@rr-research.no (K.-M.H.); Hakan.Patrik.Vernhoff@rr-research.no (P.W.); Vegard.Nygaard@rr-research.no (V.N.); sigven@ifi.uio.no (S.N.); Geir.Frode.Oy@rr-research.no (G.F.Ø.); 2 Centre for Cancer Cell Reprogramming, Institute of Clinical Medicine, Faculty of Medicine, University of Oslo, 0424 Oslo, Norway; 3 Department of Medical Biochemistry, Oslo University Hospital, Radiumhospitalet, 0424 Oslo, Norway; sigurdbo@ous-hf.no; 4 Department of Informatics, University of Oslo, 0316 Oslo, Norway

**Keywords:** melanoma, melanocyte transformation, MITF, MC1R, AXL inhibitor, familial melanoma

## Abstract

The MC1R/cAMP/MITF pathway is a key determinant for growth, differentiation, and survival of melanocytes and melanoma. MITF-M is the melanocyte-specific isoform of Microphthalmia-associated Transcription Factor (MITF) in human melanoma. Here we use two melanocyte cell lines to show that forced expression of hemagglutinin (HA) -tagged MITF-M through lentiviral transduction represents an oncogenic insult leading to consistent cell transformation of the immortalized melanocyte cell line Hermes 4C, being a melanocortin-1 receptor (MC1R) compound heterozygote, while not causing transformation of the MC1R wild type cell line Hermes 3C. The transformed HA-tagged MITF-M transduced Hermes 4C cells form colonies in soft agar and tumors in mice. Further, Hermes 4C cells display increased MITF chromatin binding, and transcriptional reprogramming consistent with an invasive melanoma phenotype. Mechanistically, forced expression of MITF-M drives the upregulation of the AXL tyrosine receptor kinase (AXL), with concomitant downregulation of phosphatase and tensin homolog (PTEN), leading to increased activation of the PI3K/AKT pathway. Treatment with AXL inhibitors reduces growth of the transformed cells by reverting AKT activation. In conclusion, we present a model system of melanoma development, driven by MITF-M in the context of MC1R loss of function, and independent of UV exposure. This model provides a basis for further studies of critical changes in the melanocyte transformation process.

## 1. Introduction

The melanocortin-1 receptor (*MC1R*) has been found to be a low-risk melanoma susceptibility gene [1,2]. *MC1R* variants have furthermore been shown to increase the melanoma risk in families possessing cyclin-dependent kinase inhibitor 2A (*CDKN2A*) mutations [3]. *CDKN2A* was identified as the first melanoma susceptibility gene more than 20 years ago, and germline *CDKN2A* mutations have been found in up to 20–40% of the melanoma-prone families worldwide [4]. *CDKN2A*-encoded p16INK4a forms a functional protein complex with CDK4, and cyclin D1, leading to phosphorylation of the RB1 protein, with subsequent impact on the G1-S control of the cell cycle. This signaling is altered in 90–100% of melanomas [5,6].

It has been proposed that a malignant transformation of melanocytes expressing *CDKN2A* mutation and *MC1R* loss-of-function allele(s) requires acquisition of somatic mutations, facilitated by the *MC1R* genotype or aberrant microenvironment due to *CDKN2A* mutation status [7]. The *MC1R* gene locus is highly polymorphic in populations of European ancestry, and more than 200 coding region variants have been identified to date, with a combined prevalence of any *MC1R* variant being present in ~60% of the population. Among these variants are the red hair color (RHC) variants associated with red hair, light skin, poor tanning ability, and heavy freckling [8]. Carriers of any MC1R variant have been shown to have a 66% higher risk of developing melanomas compared to wild-type (WT) subjects [9]. The relative impact of RHC-variants on melanoma is still being debated, as population-specific allele frequencies exist, and with differing disease outcomes [9,10,11]. Individuals of European ancestry have a higher incidence rate for cutaneous melanoma (CM) than non-Europeans, which is attributed to their fair skin type. The degree of UV protection in the skin is defined by the amount and type of pigment mediated by MC1R. UVB exposure triggers the PTEN protein interaction with WT, but not RHC-associated, *MC1R* variants, protecting PTEN from degradation, leading to AKT inactivation [12]. Functionally, the MC1R pathway normally leads to pigmentation of melanocytes through increased cytosolic cAMP, which activates the Microphthalmia-associated Transcription Factor (MITF). Therefore, RHC variant carriers exhibit reduced cAMP production, resulting in reduced eumelanin production with consequently decreased photoprotection [13]. Solar radiation exposure is deemed a common risk factor for the initiation of CM, through induction of cyclobutene pyrimidine dimers and pyrimidine (6-4) pyrimidone photoproducts (6-4PP) in DNA, leading to somatic mutations impacting cellular function [14]. However, evidence exists that melanoma also occurs in non-Sun exposed skin [15,16,17], and this argues for additional factors contributing to the development of melanoma.

In recent years, new melanoma susceptibility pathways have emerged [18], and a gain-of-function mutation detected in the microphthalmia-associated transcription factor isoform 4, or MITF-M (hereby referred to as MITF), p.E318K, has been associated with both familiar and sporadic melanoma susceptibility [19]. Carriers of this variant are associated with high nevi counts and a 3 to 4-fold increased risk for melanoma. The MC1R/cAMP/MITF pathway is implicated in growth, differentiation and survival of melanocytes, as well as in malignant melanoma [20,21]. MITF has also been shown to possess oncogenic potential in immortalized melanocytes having a BRAF V600E activating mutation [22]. Besides MITF, several high penetrance genes involved in telomere lengthening (such as *TERT*) or telomere maintenance play a role in familial melanoma predisposition [23], as well as other genes, such as *BAP1*, *POT1*, *ACD*, and *TERF2IP* [24].

Here we use the immortalized melanocyte cell lines Hermes 3C and 4C to study the non-UV-related mechanisms of melanoma initiation in the context of familial melanoma. The Hermes 3 and 4 series are immortalized melanocyte cell lines with WT and RHC *MC1R* alleles (R160W/D294H) [25,26], respectively. Hermes 3C and 4C both require melanocytic growth conditions, with normal morphologies, thus broadly retaining the in vitro culture features of normal melanocytes [25]. The Hermes cell lines recapitulate essential features of the most frequent familial high melanoma risk factors: both 3C and 4C are immortalized via ectopic expression of hTERT and inactivation of the RB1/p16/CDK4 complex through transduction of HPV16-E7. Moreover, 4C is derived from an RHC individual genetically heterozygote for *MC1R*, R160W/D294H, which results in complete loss of function of the MC1R protein, while Hermes 3C is WT for MC1R [25,26]. We show that the *MC1R* mutant Hermes 4C cell line transforms upon forced MITF expression, whilst the WT Hermes 3C does not. We present a useful model system for studying the development of melanoma in the context of defective MC1R. 

## 2. Results

### 2.1. MITF Dysregulation Induces Transformation in an MC1R-Mutated Genetic Background

**Figure 1:**
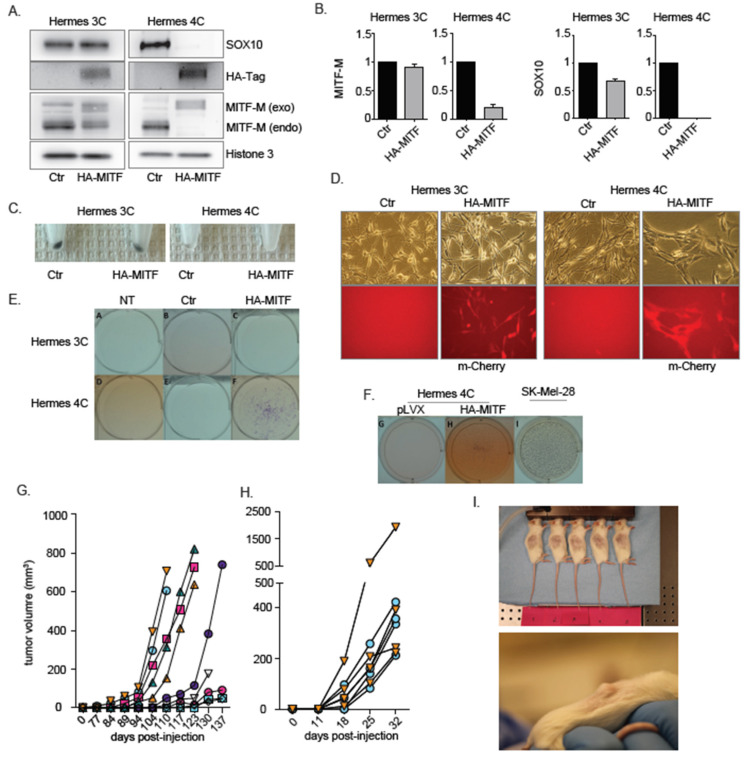
HA-MITF-M leads to transformation of immortalized MC1R mutant 4C melanocytes. (**A**) Western blot analysis of endogenous (endo) and ectopically transduced exogenous (exo) MITF-M through 3x HA Tag (HA-Tag) expression vector in 3C-HA-MITF and 4C-HA-MITF and control (Ctr) transduced lines (3C pLVX IRES mCherry and 4C pLVX IRES mCherry cells) showing the exogenous 3HA-tagged MITF, which is larger than the endogenous due to the triple HA tag. Protein levels of SOX10 are also indicated. Histone H3 was used as loading control. The image is representative of *n* = 3 experiments. (**B**) RT-PCR analysis showing (endogenous) MITF-M and SOX10 levels in 3C-HA-MITF and 4C-HA-MITF compared to 3C and 4C control cells. Graphs represents expression data from three separate experiments normalized to control and plotted as mean ± SD. (**C**) Visual inspection of pelleted 4C-HA-MITF and 3C-HA-MITF compared to 3C and 4C control cells’ pellets. (**D**) Phase contrast and fluorescent micrographs of 3C (parental control), 4C (parental control), 3C-HA-MITF and 4C-HA-MITF cells documenting the transformed morphology of 4C-HA-MITF and displaying MITF/mCherry bicistronic expression for the transduced lines. (**E**) Representative images of the growth factors independence assay for the indicated cells growing in growth factor deprived media for 21 days. NT: Non-transduced parental cells. Ctr: Control. 3C-HA-MITF and 4C-HA-MITF cells. (**F**) Representative images of the anchorage independent assay for 4C, 4C-HA-MITF and SKMEL28 cells growing in agarose. 3C and 3C-HA-MITF were not included, as they did not display growth-factor-independence. The SKMEL28 melanoma cells were used as positive control. (**G**) 4C-HA-MITF -derived tumor volume measurements in NSG mice. Each line represents a different xenograft and legends represent time of measurement. (**H**) Tumor volume measurements in NSG mice injected with newly established xenografts from 4C-HA-MITF cells at a second passage. (**I**) Representative images of NSG mice bearing 4C-HA-MITF xenograft tumors.

In order to investigate the role of MITF with respect to melanoma development in varying RHC background, HA-tagged MITF was introduced via lentiviral transduction in both the WT MC1R Hermes 3C and in the compound heterozygote MC1R R160W/D294 Hermes 4C melanocytes. The resulting stably transduced cells are hereafter referred to as 3C-HA-MITF and 4C-HA-MITF, respectively. To confirm successful transduction, we measured exogenous and endogenous MITF levels by both western blotting and RT-PCR (Figure 1A,B). We found that endogenous MITF was downregulated to undetectable levels in 4C-HA-MITF, indicating MITF expression autoregulation, while to a lesser degree in 3C-HA-MITF, when compared to their respective control transduced lines, hereafter referred as 3C and 4C (see methods). We also found that SOX10, a transcriptional activator of MITF transcription and a major cofactor of MITF in the pigmentation process was concomitantly suppressed in 4C-HA-MITF (Figure 1A,B), indicating that also *SOX10* is under MITF control.

Both SOX10 and MITF are important factors of melanocyte biology, including mediating pigmentation of melanocytes [27], and the regulation of their expression by MITF was previously suggested [28]. On harvesting the cells, we observed a loss of pigmentation in the transduced HA-MITF cells, which was more marked in 4C-HA-MITF pellets (Figure 1C), compared to 3C-HA-MITF. To investigate the cause of the pigment loss, we examined whether the inserted HA-tagged MITF protein was functionally active. We were able to detect activation of the tyrosinase promoter (an acknowledged MITF target) in 3C-HA-MITF, but not in 4C-HA-MITF (Figure S1A), by RT-PCR. We also verified the functionality of the HA-MITF protein by transducing an established osteosarcoma cell line, U2OS, known to be available for MITF-induced tyrosinase activation [29]. Transduction of U2OS cells with HA-MITF did indeed induce expression of tyrosinase transcripts, while transduction of the empty control (IRES) did not (Figure S1B,C).

Further, after lentiviral transduction of HA-tagged MITF, we also observed a transformed morphology in 4C-HA-MITF, in terms of loss of dendricity, flattening and more spread morphology, and greater clustering of cell distribution. These changes in morphology were not observed in 3C-HA-MITF cells (Figure 1D). To validate that Hermes 4C cells were indeed transformed after HA-MITF transduction, we subjected the Hermes lines and derivatives to a growth factor independence assay. Only 4C-HA-MITF cells were able to form colonies under these conditions (Figure 1E), and displayed anchorage-independence in a growth assay on soft agarose (Figure 1F). Cholera toxin imitates the growth effects of α-melanocyte stimulating hormone [30]. Growth and cell proliferation assays confirmed that HA-MITF transduction enhanced proliferation and viability of 4C, but not 3C, in the presence and absence of cholera toxin (Figure S1D–H). The transformation was reproduced four out of four times at similar frequencies.

In order to examine the possibility that the transformation could be due to other mutations possibly incurred through the experimental procedures, we performed whole-exome sequencing in both HA-MITF transduced cells and non-transduced parental cell lines. We could not identify any obvious alterations of cancer relevance in the transduced cell lines, including BRAF mutations, when compared to their respective controls. A list of identified somatic variants, and status of *CDK4*, *CDKN2A*, *MITF* and *MC1R*, in all cell lines is reported in Table S1. Next, to verify the tumorigenic potential, we xenografted Hermes 4C (parental), 4C (control transduced), and the derivative 4C-HA-MITF cells into NOD scid gamma (NSG) mice. All the mice injected with 4C-HA-MITF cells developed tumors (*n* = 10; Figure 1G), while mice injected with the control lines did not, even though they were followed for double the amount of time. The 4C-HA-MITF tumor xenografts were passaged to new mice to investigate whether it could be considered a stable line that would continue to grow. We indeed found that palpable tumors re-formed in shorter time spans after passaging than in the initial engraftment (Figure 1H–I). To ensure that the tumors were the result of outgrowth of 4C-HA-MITF transformed cells, we investigated and confirmed the levels of HA-MITF and SOX10 in the tumor xenografts by Western blotting and RT-PCR (Figure S1I–L), and *TYR* by RT-PCR (Figure S1M).

Taken together, these data suggest that forced expression of HA-MITF in the context of RHC MC1R, appears to be an oncogenic insult inducing melanomagenesis.

### 2.2. Dysregulation of MITF Alters the Melanocytic Transcriptional Program

**Figure 2:**
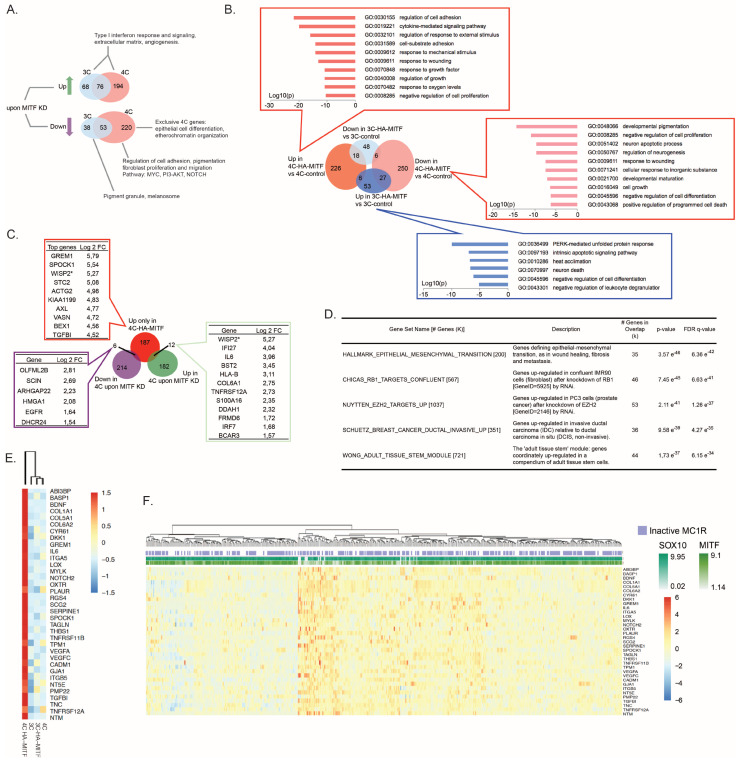
HA-MITF directs specific transcription leading to melanocyte transformation. (**A**) Differentially expressed genes (up and down regulated) upon knockdown of MITF-M in parental Hermes 3C and Hermes 4C cells. (**B**) Differentially expressed genes upon HA-MITF transduction in 3C-HA-MITF and 4C-HA-MITF cells compared to control transduced 3C and 4C cells. The boxes also show top enriched biological processes (*p* < 0.00001) according to Gene Ontology analysis. (**C**) Identification of MITF-M target genes important for Hermes 4C transformation based on overlap of genes that were down and up regulated upon MITF-M knockdown in parental Hermes 4C cells (all genes are listed in the speech bubbles along with the Log 2 fold change in 4C-HA-MITF vs. 4C control) with genes that were exclusively upregulated in 4C-HA-MITF vs. 4C control (*n* = 205; see also Table S2; top 10 upregulated genes are listed in the related speech bubble). (**D**) Gene set enrichment analysis for genes exclusively upregulated in 4C-HA-MITF cells compared to 4C control cells transduced with control vector, not concomitantly upregulated in 3C-HA-MITF vs. 3C control nor 4C control vs. 3C control (*n* = 205; see also Table S2). Clustering analysis of Hermes cells and derivatives transduced with HA-MITF (**E**) and 371 cases of melanoma from The Cancer Genome Atlas (TCGA) (**F**) according to the expression of 35 genes exclusively upregulated in 4C-HA-MITF 200 genes included in the epithelial to mesenchymal transition (EMT) hallmark signature.

In order to identify MITF-dependent genes, we performed RNA-seq of the parental Hermes 3C and Hermes 4C cells following MITF knockdown (KD) using siRNA against MITF (Figure S2A). Analysis of significantly (*p* < 0.05 and |log2 fold change| > 1) differentially expressed genes (DEGs) returned a set of 235 putative direct gene targets of MITF in the Hermes 3C line, and a set of 543 putative direct gene targets in the Hermes 4C line (Figure 2A and Table S2), of which 129 genes were overlapping. The higher number of genes affected by KD of MITF in 4C indicated that dysregulation of MITF transcriptionally affects the Hermes 4C more than the Hermes 3C cells. Of the 235 genes affected in Hermes 3C, 91 genes were downregulated, and melanosome (GO:0042470) and pigmentation regulation (GO:0043473) were among the most significantly enriched functional properties of these genes (Figure 2A and Figure S2B). The 144 upregulated genes upon MITF KD in Hermes 3C were enriched for Gene Ontology (GO) terms associated to interferon signaling response, angiogenesis, and development of neural and other adult tissue (Figure 2A and Figure S2C). Similar functional properties were also enriched within the 270 genes upregulated upon KD of MITF in Hermes 4C cells (Figure 2A and Figure S2D). In Hermes 4C cells, MITF KD induced downregulation of 273 genes with functions related to metabolic processes implicated in pigmentation, but also to protein modifications such as O-linked glycosylation and phosphorylation (Figure 2A and Figure S2E). Other genes encoding proteins within the PI3K-AKT pathway and the canonical MYC pathway were also downregulated by MITF KD in the Hermes 4C cells. Interestingly, the PI3K-AKT pathway, which is commonly activated in many cancers, including melanoma [31], seems to be under MITF regulation in Hermes 4C with the MC1R inactive variant background. Moreover, in Hermes 4C, MITF selectively maintain the expression of genes deputed to epithelial differentiation, proliferation and migration (Figure 2A and Figure S2F). Overall, the KD RNA-seq expression analysis suggests that the *MC1R* status is associated with a different MITF-regulated transcriptional program.

Next, we compared array-based gene expression data of 3C-HA-MITF and the transformed 4C-HA-MITF to their control-transduced counterparts 3C and 4C (Table S3). In agreement with our results on MITF KD in parental Hermes cells, 4C cells were most affected by the transduction of HA-MITF (Figure 2B). When comparing gene expression with their control-transduced counterparts, 4C-HA-MITF showed about four times more DEGs than 3C-HA-MITF. The genes downregulated in 4C-HA-MITF cells were enriched for melanosome formation and pigmentation, reflecting a progressive loss of cell identity and differentiation (Figure 2B) and indicating a loss of MITF canonical function in these cells. Upregulated genes in 4C-HA-MITF mostly reflected an increased metabolic activity and responses to external signaling, such as response to cytokines and cell adhesion. Gene set enrichment analysis (GSEA) retrieved gene sets such as that downregulated in the NCI60 cell lines panel in association with p53 mutation (P53_DN.V1_DN (192; *p* = 6.05 × 10^−37^), and the gene set up-regulated in fibroblasts after KD of RB1 (CHICAS_RB1_TARGETS_CONFLUENT (567; *p* = 1.89 × 10^−51^)), respectively, suggesting a possible implication of p53 function, and pRB in the process of transformation of 4C-HA-MITF. Interestingly, we could not observe a marked (log10 (*p*)< −5.0 = *p* < 0.00001) enrichment of biological processes among downregulated genes in 3C-HA-MITF vs. 3C-control, while upregulated genes showed enrichment for unfolded protein response and ER stress-related genes (Figure 2B), suggesting that 3C cells may use these pathways to avoid transformation [32].

Next, to restrict the search for important MITF target genes mediating 4C-HA-MITF transformation, we focused on genes exclusively upregulated in 4C-HA-MITF versus 4C that were not upregulated in 4C versus 3C, nor in 3C-HA-MITF versus 3C (Figure 2C). We shortlisted 205 top upregulated genes in 4C-HA-MITF (Table S3). Interestingly, some of the 205 genes were among the top-listed MITF siRNA-treated target genes, including *GREM1*, *SPOCK1*, *TGFBI*, *AXL*, *EGFR* and *IL6* (Figure 2C).

GSEA of the 205 genes revealed a marked enrichment (17.5%; *p* = 3.57 × 10^−46^) of genes encoding proteins implicated in epithelial to mesenchymal transition (EMT; Figure 2D and Table S3). Among them e.g., *GREM1*, *SPOCK1*, *TGFBI*, *AXL*, and *IL6* have all been associated with EMT [33,34,35,36,37]. Therefore, we took an unbiased approach to define whether the EMT was the process that led to the transformation of 4C-HA-MITF. First, we used a set of 200 genes defining EMT from GSEA (gene set: HALLMARK_EPITHELIAL_MESENCHYMAL_TRANSITION (200)) to cluster the control 3C and 4C cell lines and their transduced derivatives (Figure S2G). 4C-HA-MITF clustered separately from the other cell lines. A core set of 35 genes (out of the 200 genes contained in the Hallmark EMT GSEA signature) was upregulated exclusively in these 4C-HA-MITF cells (Figure 2E), suggesting that EMT is a result of the HA-MITF transduction leading to 4C transformation. In order to examine whether tumor samples could be clustered with this EMT-related gene signature, we specifically interrogated the data from The Cancer Genome Atlas (TCGA). This analysis recapitulated previous findings from Verfaillie et al. [38], displaying a distinct separation of invasive and proliferative tumor phenotypes characterized by EMT, based on expression profile [38]. One of the major determinants of the invasive subgroup was indeed the mesenchymal signature. According to the definition of the invasive phenotype by Verfaillie [38], the 4C-HA-MITF is a cell line resembling an invasive tumor (Figure S2H). Importantly, the TCGA analysis revealed a high frequency of MC1R mutated tumors, as previously suggested in other cohorts [39]. Taken together these data suggest that HA-MITF in the context of MC1R loss of function induces upregulation of direct MITF targets of EMT-related genes, in this way inducing oncogenic transformation with subsequent acquisition of invasive properties.

### 2.3. MITF Binding to the Chromatin in Melanocytes Carrying MC1R-Loss of Funtion Variants Is Increased Compared to Wild-Type MC1R Carrying Melanocytic Cells

Given the transformation of 4C cells when transduced with HA-MITF, we hypothesized that altered MITF binding to chromatin might play a role in the transformation process. To this end, we performed a chromatin immunoprecipitation followed by sequencing analysis (ChIP-seq). The reason for utilizing an HA-tag in our construct was that high-quality antibodies specific for the MITF-M protein isoform are still lacking. We therefore utilized the HA-tag to ensure antibody-specificity to the MITF-M isoform.

**Figure 3:**
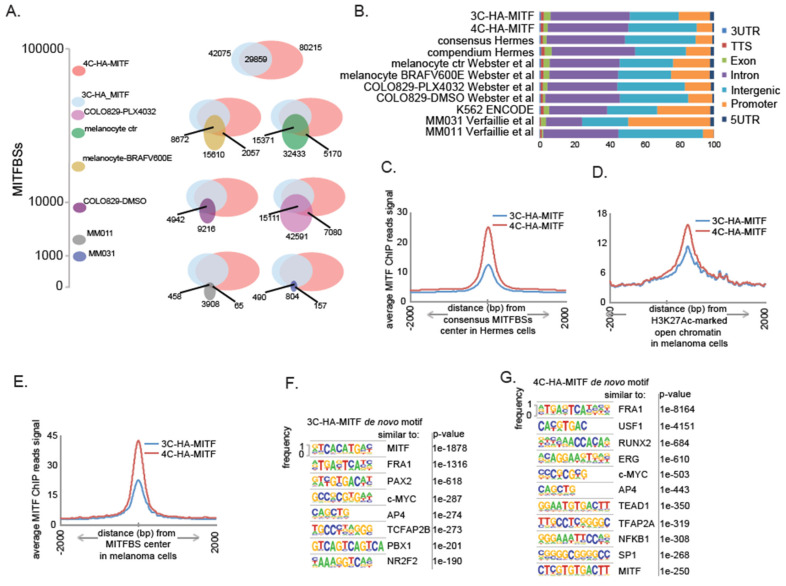
HA-MITF-M enhances the chromatin binding and reprograms transcription. (**A**) MITF binding sites (MITFBSs) number in Hermes 3C-HA-MITF and Hermes 4C-HA-MITF and overlap with the indicated datasets. MITFBSs in COLO829 and Melanocytes data are from Webster et al. [41], MITFBSs in Melanoma cell lines MM011 and MM031 are from Verfaillie et al. [38]. (**B**) Distribution of MITF binding sites (MITFBSs) according to annotated genomic region. From this study and publicly available datasets as indicated. (**C**) Distribution analysis of MITF ChIP-seq reads in 3C-HA-MITF and 4C-HA-MITF cells around consensus MITF binding sites (MITFBSs) in Hermes cells. (**D**) Distribution analysis of MITF ChIP-seq reads in 3C-HA-MITF and 4C-HA-MITF cells around high confidence open chromatin marked with H3K27Ac in 12 melanoma cell lines from Verfaillie et al. [38] (see Methods). (**E**) Distribution analysis of MITF ChIP-seq reads in 3C-HA-MITF and 4C-HA-MITF cells around high-confidence MITFBSs retrieved from the melanoma cell line COLO829 and transformed Melanocyte through forced expression of BRAFV600E from Webster et al. [41] (see Methods). (**F**) De novo motif discovery analysis using 42,075 MITFBSs’ DNA sequences retrieved from 3C-HA-MITF cells. (**G**) De novo motif discovery analysis using 80,215 MITFBSs’ DNA sequences retrieved from 4C-HA-MITF cells.

We performed two biological ChIP-seq experiments in each cell line and identified 42,075 high confidence (common to both experiments) MITF-M binding sites (MITFBSs) in 3C-HA-MITF cells, and almost twice as many (80,215 high confidence sites) in 4C-HA-MITF cells. The MITFBSs overlapped to a significant extent between the two cell lines, as approximately 70% of the MITFBSs in 3C-HA-MITF were also found in 4C-HA-MITF (Figure 3A), and therefore we defined them as consensus MITFBSs, as opposed to the union of all MITFBSs found in the two cell lines, which we called compendium MITFBSs.

To evaluate the quality of the MITFBSs observed in the Hermes cell lines, we compared our MITF-M ChIP-seq data with data from previously published data [40] (Figure 3A). Consensus MITFBSs overlapped 40–60% with these datasets. We further noted that less than 30% of the MITF bindings sites found in the three melanoma cell lines COLO829 [41], MM031, and MM011 [38], for which public data on MITF Chip-seq were available, overlapped. Around 50% of the MITFBSs have been reported to locate in a 10 Kb segment around the transcription start sites (TSSs) in the melanoma cell line 501Mel [42], while more distant MITF bound enhancers, such as one −67 Kb away from the MET gene, have also been shown to be clinically relevant [41]. In line with previously published results on melanoma cell lines and melanocytes [38,41,43], we in both Hermes cell lines found that MITFBSs present mainly in intronic and intergenic regions, but also in promoter-proximal regions 1Kb around the TSSs (Figure 3B). The upstream or downstream region of 70% of the genes modulated by transduction of HA-MITF in Hermes cells had at least one MITFBs within 25 Kb (Table S2). The binding of HA-MITF was nevertheless enhanced in 4C-HA-MITF cells, with multiple MITFBSs in proximity of several of these cancer-relevant genes (e.g., *AXL*, *IL6*, *TGFBI*, and *EGFR*,) compared to HA-MITF binding in 3C-HA-MITF (Figure S3A–D). We further found that HA-MITF was bound to the promoter regions of both the *MITF* and *SOX10* genes in both cell lines (Figure S3E,F). The data suggest that these loci are under complex regulation by MITF in both cell lines, and support the recent findings that both *MITF* and *SOX10* are transcriptionally regulated by MITF-M [44]. Based on these observations, we proceeded to validate also the expression changes for the MITF-dependent genes *AXL*, *IL6*, *TGFBI*, *SPOCK1*, and *GREM1* in the Hermes cell lines, via RT-PCR (Figure S3G). Accordingly, a distribution analysis of the MITF ChIP-seq reads around the MITFBSs consensus (Figure 3C) and compendium (Figure S3H) in Hermes cells (see methods) indicated enhanced chromatin binding of MITF in 4C-HA-MITF.

### 2.4. MITF Binding Is Associated with Open Chromatin

To examine whether MITFBSs could be associated with chromatin structure as mapped in a melanoma setting, we compiled high-confidence maps (see Methods) of open genomic regions enriched for H3K27Ac and H3K27me3 marks, indicating active or repressed chromatin, respectively, from twelve melanoma cell lines from published studies [38] and performed the same distribution analysis of MITF ChIP-seq reads in 3C- and 4C-HA-MITF cells. We found that MITF chromatin binding in 4C-HA-MITF is enhanced preferentially in open regions associated with active transcription, as marked with H3K27Ac (Figure 3D and Figure S3I,J), as opposed to regions marked with the repressive mark H3K27me3 (Figure S3K). We also compiled high-confidence MITFBSs maps from the three melanoma cell lines COLO829, MM011, and MM031, having constitutively active expression of BRAFV600E [38,41]. A distribution analysis of MITF ChIP-seq reads around these mapped sites revealed an enhanced MITF binding in 4C-HA-MITF cells (Figure 3E and Figure S3L).

A motif-enrichment analysis was undertaken for both 3C and 4C -HA-MITF cells consensus MITFBSs (Figure S3M,N). The main 3C-HA-MITF-binding patterns related to the E-box-binding patterns [45], while the 4C-HA-binding patterns related to AP-1 transcription factors, including FRA1 (FOSL1), FOSL2, and JUN-AP1.

In a de novo motif discovery analysis looking for motifs of length between 6 bp and 18 bp, we again found that in 3C-HA-MITF cells, the E-box motifs were the top enriched motifs (Figure 3F). In 4C-HA-MITF, other binding motifs were more prominent, notably factors previously reported to be involved in phenotype switching to an invasive phenotype having low MITF expression, such as FRA1 (FOSL1), TEAD, NFKB1 (Figure 3G) [38,46]. Taken together, these results indicate that transformed melanocytes with MC1R-loss of function variants display an enhanced MITF-binding to the chromatin compared to MC1R WT-carrying melanocytic cells. However, these results do not delineate the sequence of events controlling the observed transformation.

### 2.5. MITF-Dependent Dysregulation of AXL Contributes to Altered Proliferation of 4C-HA-MITF Cells

**Figure 4:**
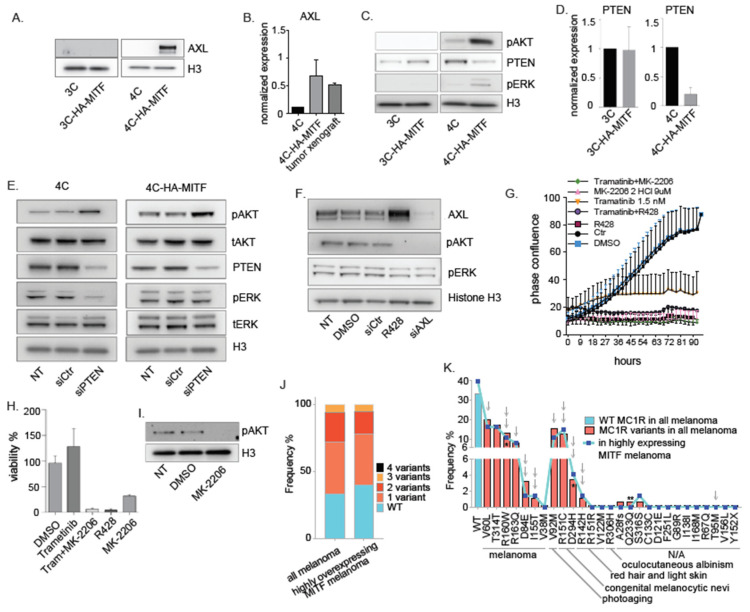
AXL and PI3/AKT pathway mediates HA-MITF transformation through PTEN downregulation. (**A**) Western Blot analysis of AXL in 3C-HA-MITF and 4C-HA-MITF compared to parental cell lines. Histone H3 was used as loading control. Representative blot from three different experiments. (**B**) Graphs represent RT-PCR expression data comparing AXL levels in 4C-HA-MITF, and in 4C-HA-MITF cells xenografted in mice normalized to 4C cells and plotted as mean ± SD (*n* = 3). (**C**) Western Blot analysis of pAKT, PTEN and pERK after cholera toxin starvation in 3C-HA-MITF and 4C-HA-MITF normalized to 3C and 4C levels. Histone H3 was used as loading control. Representative blot from three different experiments. (**D**) Graphs represent RT-PCR expression data comparing PTEN levels in 3C-HA-MITF, and in 4C-HA-MITF cells normalized to control 3C and 4C cells and plotted as mean ± SD (*n* = 3). (**E**) Western Blot analysis of the indicated proteins levels after PTEN depletion in the parental 4C cell line and transduced 4C-HA-MITF compared to scrambled siRNA and non-treated control (NT). Histone H3 was used as loading control. Representative blot from three different experiments. (**F**) Western blot analysis of AXL, pAKT and pERK in 4C-HA-MITF treated with AXL inhibitor R428 (2 μM) and siAXL compared to non-transduced (NT), scrambled siRNA and DMSO control. Histone H3 was used as loading control. Representative blot from three different experiments. (**G**) Growth curves measured using IncuCyte of 4C-HA-MITF cells untreated (NT), or control treated with DMSO (DMSO), or MK-2206 (9 μM), Trametinib (1.5 nM), R428 (2 μM), a combination of Trametinib (1.5 nM) with MK2206 (9 μM), and a combination of Trametinib (1.5 nM) and R428 (2 μM) (*n* = 3). (**H**) Graph represents cell viability measurements using MTS of 4C-HA-MITF after 90 h treatment with the indicated inhibitor treatments normalized to DMSO treated cells and plotted as mean ± SD (*n* = 3). (**I**) Western blot analysis of testing the efficacy of the MK-2206 inhibitor in 4C control. (**J**) Frequency of MC1R co-occurring mutations in the TCGA melanoma cohort (*n* = 471), and in 73 melanoma cases from the same cohort that displayed high expression of MITF (cut off = Z-score ≥ 1). (**K**) Frequency of the indicated MC1R mutations in the TCGA melanoma cohort (*n* = 471) and in the 73 cases with high expression of MITF. Their associated phenotypes according to [48,49,50] are indicated (N/A is not applicable or not known) as well as whether the variant is highly (double arrow), or mildly (single arrow) impairing MC1R function. * indicates the MC1R variants co-occurring in Hermes 4C cell lines; ** indicates the synonymous mutation of the MC1R that does not alter the amino acid sequence of MC1R.

AXL was one of the top upregulated genes in 4C-HA-MITF cells (Figure 2C and Figure 3C). Previous studies have implicated the activation of the AKT pathway by AXL in melanoma [47]. To examine how MITF dysregulation could impact the increased proliferative potential of the 4C-HA-MITF cells, we first confirmed selective AXL upregulation in the transformed 4C-HA-MITF cells at the protein level (Figure 4A). AXL was also upregulated in 4C-HA-MITF xenografts at the mRNA level (Figure 4B) and protein level (Figure S1I). We hypothesized that AXL upregulation would result in AKT activation also in our melanoma transformation model. By western-blot analysis, we confirmed that under cholera toxin-starved conditions, 4C cells, and to a greater extent 4C-HA-MITF cells, displayed higher basal levels of pERK and pAKT, compared to the 3C and 3C-HA-MITF cell lines (Figure 4C)), which could be seen also in the xenografted 4C-HA-MITF (Figure S1I). The expression data also revealed that PTEN, a known suppressor of the PI3K/AKT pathway, showed lower expression in 4C-HA-MITF compared to 4C (Table S2). Accordingly, we found that PTEN was downregulated in 4C-HA-MITF compared to 4C at the protein (Figure 4C) and mRNA level (Figure 4D), while this was not the case in 3C-HA-MITF versus 3C cells. To examine whether PTEN downregulation could directly increase pAKT, we depleted PTEN by siRNA transfection in the 4C cell lines. As expected, 4C and 4C-HA-MITF responded with an increase in pAKT phosphorylation upon PTEN depletion, demonstrating a repressive effect of PTEN upon PI3K signaling (Figure 4E).

Next, we targeted AXL by an AXL siRNA and an AXL inhibitor (R428) to assess whether this could counter the increased proliferation observed in 4C-HA-MITF cells. Depletion of AXL by the siRNA and inhibitor abolished the phosphorylation of AKT, while only the siRNA reduced the AXL level (Figure 4F). Surprisingly, the inhibitor seemed to increase the expression of AXL. AXL inhibition alone led to the greatest reduction of cell proliferation and viability (Figure 4G,H). To explore whether the decrease in cell proliferation and viability was due to pAKT reduction itself or other possible effects of the AXL inhibitor, we used an AKT inhibitor (MK-2206) and a MEK inhibitor (trametinib, Figure 4I,G, respectively), which proved highly effective in inhibiting AKT-induced growth and survival of 4C-HA-MITF cells (Figure 4G,H). We found that inhibiting the MAPK pathway using Trametinib was less effective than targeting the PI3K-AKT pathway using the AKT inhibitor MK-2206.

### 2.6. Defective MC1R and MITF Overexpression Co-Occur in Malignant Melanoma

As transformation occurs in the *MC1R*-variant Hermes 4C cell line, but not in the WT Hermes 3C cell line, we examined TCGA containing a cohort of 470 melanoma cases of Skin Cutaneous Melanoma (SKCM), for the expression status of MITF in *MC1R* variant carriers. We found that 10–15% of the 470 tumors had relatively high (Z-score > 1) expression of MITF (Figure S4). In agreement with previous studies, more than 60% of the 470 samples presented with variants of MC1R. We found no significant association between MITF overexpression and MC1R inactivation. However, MITF overexpression and MC1R inactivation co-occur in 38.3%, 16.4%, and 5.4% of the highly expressing MITF which presented also a *MC1R* gene with at least one, two, or three mutations, respectively (Figure 4J and Table S4). Of note, the same *MC1R* mutation in 4C R160W/D294H occurred five times in the whole dataset and one time in the MITF overexpressing tumors. However, the most frequent variants in the MC1R gene were highly inactivating and reflected variants associated prevalently with risk of melanoma, photoaging, and with RHC phenotype (Figure 4K).

## 3. Discussion

The mechanisms behind oncogenesis in melanoma are currently not well understood, as the availability of relevant experimental models is limited. We present here a melanomagenesis model based upon immortalized melanocyte cells that recapitulate features of heritable melanoma to study the development of melanoma in an RHC setting. In our model, we find that the lineage-specific master regulator of melanocyte development and survival, MITF, acts as an oncogene when transduced in the form of HA-MITF in an hTERT/RB1/CDK4/p16/MC1R RHC background. Context-dependent oncogenic MITF was first described by Garraway and colleagues as an event that required dual transduction of BRAFV600E and HA-MITF in an hTERT/p53DD/CDK4(R24C) background [51]. Later, it has been suggested that BRAFV600E-transduction alone is sufficient for transformation of hTERT/p53DD/CDK4(R24C) human melanocytes depleted for MC1R, in that MC1R WT protects PTEN protein against degradation after UVB-exposure [12]. Loss of this function in the form of MC1R RHC, followed by degradation of PTEN, is associated with enhanced PI3K/AKT pathway signaling [12]. Epigenetic, mutational and deletion events are believed to account for PTEN dysregulation in as many as 40–50% of sporadic melanomas [52]. The PI3K/AKT pathway has been found to disrupt BRAFV600E-induced senescence [12], which further emphasizes an oncogenic role of this signaling pathway within melanoma initiation and development.

In the model presented here, HA-MITF transduction alone is sufficient to induce transformation of 4C cells. The transformed RHC MC1R melanocytes display increased MITF-binding to the chromatin compared to MC1R WT-carrying melanocytic cells. Moreover, MITF-binding reflects aberrant chromatin structure observed in melanoma [38]. The binding distribution of MITF coincides with DNA motifs of TFs involved in melanoma development such as SOX10, MITF, AP-1, TEAD and others. In agreement with Verfaillie et al. and others [38,53], we also observe a concomitant transcriptional reprogramming with subsequent acquisition of invasive properties after the loss of endogenous transcription factors SOX10/MITF and gain of FOS/FOSL (members of AP-1). Interestingly, our array-based gene expression data (Table S3) indicated a high upregulation of FOSL1 in 4C-HA-MITF compared to control transduced 4C. Moreover, parental 4C showed 3-fold higher JUN expression than its wild type 3C counterpart. Post-transduction JUN expression is further increased as are the AP-1 members FOS and FOSL1. These observations, together with those previously described by others suggest that AP-1 up-regulation may play a role in the observed transformation [38,54,55]. MITF and AP-1 have also been implicated in reciprocal regulation of each other [38]. Alternatively, and also in agreement with previous reports [28], our study suggests MITF directed negative feedback transcriptional control of both SOX10 and MITF expression could possibly be involved in the oncogenic transformation.

The role of HA-MITF behind the transformation process needs further clarification. However, in agreement with previous reports [28], our study suggests a negative feedback loop controlling transcriptionally the expression of both SOX10 and MITF. The transition from a proliferative towards an invasive phenotype was further characterized by upregulation of relevant EMT genes such as AXL. We identified PTEN downregulation and up-regulation of AXL receptor and its cognate ligand GAS6, all modulators of the PI3K/AKT signaling pathway [56]. Consistently, we find activation of AKT, and to a lesser extent ERK. Moreover, growth and proliferation of the transformed cells is effectively disrupted by the use of AKT or AXL inhibitors, giving further support for PI3K/AKT signaling pathway as the main driver of proliferation.

Phosphorylation of PRS6 through mTOR activation has been shown to be a marker for malignant transformation in melanocytic lesions downstream of PI3K/AKT [57]. It may provide an avenue for further research.

It has previously been reported that pharmacological downregulation of MC1R in MC1R-proficient melanocytes is associated with increased PI3K/AKT activation, possibly via MITF [58]. Our findings suggest that 4C-HA-MITF transformation is highly dependent on transcriptional upregulation of AXL by HA-MITF, and that this leads to activation of the PI3K/AKT pathway, while this happens in the context of MC1R-defective melanocytes.

MITF is amplified in circa 20% of melanoma tumors, and this is thought to contribute to tumor progression [51]. It is also known that the germline variants of MC1R that inactivate or reduce the function of the receptor have been linked to an increased risk of developing melanoma, especially under UV exposure [8,59]. The frequency of MC1R variant carriers in the general population is approximately 67% [9,60,61] and seems also to be the case in melanoma patients [8]. Our query of the TCGA returned no significant correlation for high MITF expression and MC1R variants. This suggest that the model presented here could represents a low MITF invasive phenotype. Regardless, we observed a high co-occurrence of MC1R variants and high MITF expression in melanoma patients (up to 38% co-occurrence), indicating that a subset of melanoma may be driven by mechanisms similar to those reported for the model presented here. These findings have implications for our understanding of melanomagenesis and for identification of individuals at higher risk of developing melanoma.

## 4. Methods

### 4.1. Cell Lines and Culture Conditions

The immortalized melanocyte cell lines Hermes 3C and 4C were purchased from the Wellcome Trust Functional Genomics Cell Bank (St George’s University of London, London, UK). To ensure melanocyte proliferation, cholera toxin was added to the media as recommended by Wellcome Trust. CT stimulates cyclic adenosine 3′,5′-monophosphate (cAMP) production within the cells. All Hermes lines and their derivatives were cultured as previously described [40], with the following modifications: RPMI was replaced by M254 medium (Cascade Biologics, Portland, OR, USA) and cells were incubated in 5% CO_2_. SKMEL28 was purchased from ATCC and grown in RPMI (Sigma Aldrich, Saint Louis, MO, USA) with 8% FBS (PAA), 5% CO_2_, at 37 °C. U2OS cells were obtained from ATCC and grown in RPMI (Sigma Aldrich) with 8% FBS (PAA), 5% CO2, at 37 °C. Further details about cloning, constructs, and lentiviral production, culture conditions, growth factor and anchorage independence assays, as well as siRNA experiments, isolation of RNA, and establishment of tumor xenografts are provided in the Supplementary Methods.

### 4.2. Generation of Amino Terminal 3xHA Tagged MITF Hermes Melanocytes

Hermes 3C and Hermes 4C melanocytes were subjected to lenti-particle transduction of pLX 3xHAvar4mCh (vector expressing 3xHA tagged MITF-M (variant 4)) or control vector pLVX IRES mCh at a MOI of <1. After 5 passages, the cells were sorted for the expression of bicistronic mCherry, and allowed to expand further. Stable cell lines were subsequently re-sorted based on the presence of mCherry and used further. Cell sorting was performed by flow cytometry at the Oslo University Hospital Core Facility. This process resulted in four cells lines which we called 3C and 4C, for control vector transduced cells, and 3C-HA-MITF and 4C-HA-MITF, for cells transduced with the vector expressing 3xHA tagged MITF-M.

### 4.3. Western Blot Analysis

Western Blot analysis was performed as previously described [40]. The following antibodies were purchased from Cell Signaling Technology (Danvers, MA, USA); MITF (1:1000; #12590), SOX10 (1:1000; #14374, Phospho-Akt-Serine-473 XP (1:2000; #4060), Phospho-ERK–Thr202/Tyr204) XP (1:3000; #4370), PTEN XP (1:1000; #9188) and Histone H3 (1:3000; #4499) was used as loading control. Anti-HA-tag (12CA5) was purchased from Roche. Secondary antibody against rabbit (1:5000; P0448) was purchased from Dako (Agilent Technologies, Glostrup, Denmark). Uncropped, unedited blots from Figure 1A, Figure 4A,C,E,F,I as well as for Figure S1I and Figure S2A can be found in Figure S5.

### 4.4. Animal Experiments Approval

Animal experiments were approved and performed as stated by the Norwegian Animal Authority (Permit number 12080), and conducted according to the regulations of the Federation of European Laboratory Animal Science.

### 4.5. Establishment of Xenografts

NSG mice were anesthetized with 3% Sevoflurane (Baxter) and subcutaneously injected on both flanks with five million Hermes 4C (parental), Hermes 4C transduced with pLVXIRESmCh (vector control transduced 4C cells) and Hermes 4C cells transduced with pLX3xHAvar4mCh vector (4C-HA-MITF) in 200 µL RPMI1640. Tumor volume and body weight was registered once a week. Further details are provided in the Supplementary Methods.

### 4.6. Chromatin Immunoprecipitation

3C-HA-MITF and 4C-HA-MITF cells were used for the Chromatin immuno-precipitation with sequencing (ChIP-seq) experiments in two replicates, essentially as described in Alver et al., [40], using anti-HA-tag (12CA5) for immuno-precipitation of HA-tagged MITF.

### 4.7. Quantitative Reverse Transcriptase PCR

Quantitative real time PCR assays were performed as previously described [40]. Primers used for for Real-time (*MITF-M*, *SOX10*, *TBP*, *RPLP0*, *AXL*, *SPOCK1*, *GREM1* and *IRES*) were purchased from Integrated DNA Technologies (IDT, Coralville, IA, USA). Primers for *PTEN*, *TGFBI* and *IL6* were purchased from Bio-Rad Laboratories, Inc. (Hercules, CA, USA). All sequences are given in Table S5.

### 4.8. ChIP, RNA, and Exome Sequencing, Expression Microarray Hybridization, and Bioinformatics Analyses

ChIP-seq analysis was performed as previously described [62] with minor modifications as described in Supplementary Methods. Peak calling was performed against input, and bigwigs were normalized. Read distribution was performed with bwtool [63] with averaging of both replicates and normalizing against input sequences. Motif analysis was performed with de novo discovery settings looking at motifs from 6 bp to 18 bp, and with peaks annotations on genomic features as obtained with the HOMER package v4.10 [64].

High-confidence maps of genomic regions enriched for H3K27Ac (36,357 sites), H3K27me3 (49,821 sites), and open chromatin (11,065 sites) were compiled with Bedtools [65], intersecting ChIP-seq data for H3K27Ac, H3K27me3 and Formaldehyde assisted isolation of regulatory elements (FAIRE)-seq datasets of 12 melanoma cell lines (MM001, MM011, MM031, MM034, MM047, MM057, MM074, MM087, MM099, MM117, MM118, and SKMEL5) from Verfaillie et al. [38]. A separate map was generated intersecting consensus H3K27Ac sites with consensus open chromatin sites (5170 sites). Two maps of high-confidence MITFBSs in primary transformed and melanoma cell lines were retrieved by intersecting MITFBSs in transformed melanocytes with enforced expression of BRAFV600E and melanoma cell lines COLO827 (treated with DMSO condition only) from Webster et al. [41]. This MITFBS had 4023 sites. A second high-confidence MITFBSs map (234 sites) was build intersecting MITFBSs retrieved in the melanoma cell lines MM011 and MM031 from Verfaillie et al. [38].

RNA-seq data on MITF knockdown in 3C and 4C cells were analyzed using STAR (v. 20201) with default parameters for alignment to hg19. The resulting bam files were then fed into cuffdiff (v 2.2.1) using all conditions to build a base for transcript analysis.

Gene ontology and pathway analysis were performed using geneMANIA [66], MetaScape [67], and GSEA [68] accessed online in Sept 2018, and all ontologies and pathways are *q* < 0.05. Mutation analysis from whole-exome sequencing and microarray analyses are described in Supplementary Methods. All data are deposited in GEO under the accession number GSE142018.

### 4.9. Statistical Analysis of Melanoma Cohorts

RNA-seq expression data from the TCGA-SKCM cohort [69], measured as transcripts per million (TPM), were downloaded from cBioportal [70,71]. Heatmaps and clustering of Z-scores were performed on log2(TPM + 1) transformation in a heatmap (NMF package, version 0.21.0) [72] using R version R 3.5.0.

Alignment files (BAM) for the RNA-sequenced samples (*n* = 471) were obtained from the Genomic Data Common Data Portal using manifest download verification [69].

The MC1R mutations were analyzed by extracting sequences for MC1R gene region from BAM files, using the samtools view and mpileup options (version 1.9) [73]. Pooled VCF (variant calling format) files were constructed in BCF tools (version 1.8-41) by the normalizing, filtering, and merge options. Annotation of the MC1R variants was performed in ANNOVAR [74], using humandb hg38 (Ensembl) [75], by filtering against known mutations in the databases: refGene, exac03, avsnp147, dbnsfp30a, cosmic81, ALL.sites.2015_08. Annotated MC1R variants were summarized using vcf2table [76] and added as phenotypic information to heatmaps.

## 5. Conclusions

Here we propose an HA-MITF-induced model of melanomagenesis based upon the innate oncogenic properties of MC1R RHC, representing enhanced risk for PI3K/AKT pathway activation and oncogenic transformation in families possessing CDKN2A loss of function. Our study opens the path for further investigations on the potential oncogenic role of MITF-M as a molecular switch in MC1R RHC backgrounds.

## Supplementary Materials

The following are available online https://www.mdpi.com/2072-6694/12/7/1719/s1, Figure S1. HA-MITF-M leads to transformation of immortalized MC1R mutant 4C melanocytes, Figure S2. HA-MITF directs specific transcription leading to melanocyte transformation, Figure S3. HA-MITF-M enhances the chromatin binding. Representative UCSC genome browser snapshot showing enrichment of MITF-M binding in proximity of EGFR, Figure S4. MITF-M and MC1R in the TCGA dataset. Expression analysis of MC1R and MITF genes in the 471 tumors from the TCGA melanoma cohort, Figure S5. Uncropped blots relative to Figure 1A, Figure 4A,C,E,F,I and to Supplementary Figure S1I, and Figure S2A, Table S1. Summary of Mutations in hermes cells, Table S2. RNA-seq analysis of differentially expressed genes in 3C and 4C cells upon MITF-M knockdown, Table S3. Expression array analysis in Hermes 3C and 4C control cells and their derivative 3C-HA-MITF and 4C-HA-MITF. The values are Log2 of foldchanges in the indicated comparisons, Table S4. 471 melanoma cases with clinicopathological information from TCGA with gene expression data for MC1R and MITF, and mutations relative to the MC1R gene, Table S5. Primers and Oligos sequences.
