# Supplementary material for: Dysregulation of MITF Leads to Transformation in MC1R-Defective Melanocytes
Source: Cancers (Basel). 2020 Jun 28;12(7):1719. doi: 10.3390/cancers12071719 (PMC7408466; doi:10.3390/cancers12071719)
Supplement: Supplementary file 1 [file cancers-12-01719-s001.zip › cancers-830323 supplementary_checked.docx]

**Supplementary Materials:**

Dysregulation of MITF Leads to Transformation in MC1R-Defective Melanocytes

Timothy J. Lavelle , Tine Norman Alver, Karen-Marie Heintz, Patrik Wernhoff, Vegard Nygaard, Sigve Nakken, Geir Frode Øy, Sigurd Leinæs Bøe, Alfonso Urbanucci and Eivind Hovig

1. Supplementary Methods

1.1. Construction of Plx3xhavar4mch Vector Expressing 3xha Tagged MITF-M

The construction of pLX 3xHAvar4mCh was performed by two sequential clonings. The human MITF variant 4 cDNA was prepared by PCR amplification of the cDNA clone MGC:75121 (IMAGE:6066096) (Invitrogen, Carlsbad, CA, USA) using the BglII containing MITF forward primer:5′-AA AAAAAGATCTACCATGCTGGAAATGCTAG-3′ and the EcoRI containing reverse primer: 5′-AAAAAGATTTCATCTCGCTAACAAGTGTGC. Subsequent to restriction digestion by BglII/EcoRI (NEB), the MITF variant 4 cDNA containing insert was gel purified using the JetStar Gel extraction Kit (Genomed, Warsaw, Poland). The murine MITF variant 4 cDNA was removed from pCMV3 x HAvar4 plasmid by BglII/ EcoRI endonuclease digestion (NEB) [1] and the vector backbone containing the 5´ 3 x HA tag was isolated by gel extraction as described above. The insert and vector were then ligated overnight at 14 °C using T4 DNA ligase (NEB), transformed into chemically competent DH5α (Invitrogen) and selected candidates were screened by PCR for the presence of the insert. The successful construct pCMV3 x HAvar4 was digested with NheI/XbaI (NEB) and the 5′ 3 x HA tagged MITF variant 4 fusion insert was isolated by gel purification. The insert was then ligated into gene cleaned (Genomed) SpeI linearized pLVX IRES mCh (Clontech, Mountain View, CA, USA) producing the Lenti-expression vector pLX3xHAvar4mCherry and transformed into chemically competent STBL3 (Invitrogen). pLX3xHAvar4mCherry was subsequently sequenced (Eurofins, Luxembourg, Luxembourg) utilizing the commercially available pCMV_F(21 mer) MWG-Biotech AG and IPCR IRES 1R primers to confirm the correctness of the PCR derived human MITF variant 4 cDNA insert.

1.2. Production of Lentiviral Particles

Lentiparticles were produced in Lenti-X 293T (Clontech) using the designated lenti-expression vector and the second-generation packaging system utilizing pCMV-dR8.2 dvpr and pCMV-VSV-G as previously described [2] with the following modification: The transfection reagent Fugene (Promega, Medison, WI, USA) was replaced by Turbofect (Thermo Fisher Scientific, Waltham, MA, USA) and applied as recommended by the manufacturer

1.3. Growth Factor Independence Assay

Cells were seeded out in Nunc 6-well plates (Corning, Tewksbury, MA, USA) at a density of 2.5 × 10^4^ cells per well in RPMI-1640 Medium (Sigma-Aldrich, Saint Louis, MO, USA) supplemented with 0.5% FBS (PAA) and incubated at 37 °C and 5% CO_2_ for 21 days. Medium was replaced every 4–5 days until the completion of the assay. At completion, cells were washed twice with ice-cold PBS before a 10 min. fixation by the addition of ice-cold methanol. Staining was achieved by the addition of 0.5% crystal violet solution at room temperature for 10 min, followed by washing in ddH2O until only cell-retained dye remained. Plates were then dried overnight and photographs were captured on a Nikon D5200 using a 28 mm lens.

1.4. Anchorage Independence Assays

2.5 × 10^4^ cells were inoculated in 1 mL 0.4% agarose RPMI supplemented with 8% FBS and plated in triplicate on 0.6% agarose RPMI supplemented with 8% FBS and penicillin/streptomycin for 28 days at 37 °C and 5% CO_2_. Cells were fed every 4–5 days.

Cells were grown for 21 days in 0.3% LGT agarose RPMI 10% FBS supplemented with antibiotics. Hermes4C and the metastatic melanoma cell line SKmel28 was used as negative and positive control, respectively.

Cells were plated at a density of 2.5 × 10^4^ cells per well in 0.3% LGT agarose RPMI supplemented with 10% FBS and antibiotics. Plates were then incubated at 37 °C and 5% CO_2_ 21 days with the addition of fresh medium every 4 to 5 days. Upon completion, 400 µL NTB (1 mg/mL) was added and the plates were incubated overnight under culture condition as described. Photographs were captured on a Nikon D5200 using a 28 mm lens.

1.5. Growth Curve

Approximately 800 cells were seeded in 96-well plates and growth was followed with the IncuCyte system (Essen Bioscience, Ann Arbor, MI, USA). When indicated, cells were starved of cholera toxin for 3 days after which media containing the appropriate concentration of cholera toxin was replaced.

1.6. Establishment of Xenografts

Animal experiments were approved and performed as stated by the Norwegian Animal Authority (Permit number 12080), and conducted according to the regulations of the Federation of European Laboratory Animal Science (FELASA). NOD scid gamma (NSG) mice (NOD.Cg-Prkdcscid Il2rgtm1Wjl/SzJ) were locally bread and housed according to the guidelines at the Section of Comparative Medicine, the Norwegian Radiumhospital. Briefly, the mice were housed in open cages with free accesses to food and water. The environmental parameters in the animal rooms were as follows; 12 hours light/dark cycle, constant temperature of 21+/− 2C, a relative humidity of 30–70% and approximately ten air changes every hour. For establishment of xenografts, NSG mice were anesthetized with 3% Sevoflurane (Baxter, Deerfield, IL, USA) and subcutaneously injected on both flanks with 5 million of Hermes 4c (parental), Hermes 4c transduced with pLVXIRESmCh (vector control transduced 4C cells) and Hermes 4c cells transduced with pLX3xHAvar4mCh vector (4C-HA-MITF) in 200 µL RPMI1640. Tumor volume and body weight was registered once a week. The diameter of the tumors was measured using a caliper, and tumor volume was calculated as follows: V = w^2^ × L × 0.5 (were w and L are the shortest and longest tumor diameter respectively). The mice were sacrificed when tumor volume reached approximately 800–1000mm^3^, and tumor pieces (2 x 2mm) were passaged on to new animals.

1.7. Cell Culture Imaging

2.5 × 10^4^ cells per well were plated in 6-well plates for micrographic documentation of Hermes 3C, Hermes 4C and their pLXHA-MITF mCherry and pLVX IRES mCherry derivatives. The micrographs were captured using the 10× objective for phase contrast (white light) and Texas Red filtered (UV) fluorescent imaging on a Zeiss S200 fluorescent confocal microscope using Axiovision software v2006 (Carl Zeiss Microscopy, LLC, White Plains, NY, USA).

1.8. siRNA Experiments

5 x 10^4^ cells per well were plated in 6-well plates seeded in 6-well plates and grown to 60% confluence before being transfected using siRNA directed against MITF-M (Eurogentec, Seraing, Belgium). The cells were incubated for 72 h to ensure optimal target reduction at the protein level. The cells were transfected with a final concentration of 25 pmol siRNA, using Lipofectamine RNAiMAX (Invitrogen) as described in the manufacturer’s protocol. Sequences for siRNA are described in supplementary methods. All transfections were performed in triplicate.

1.9. Isolation of RNA

Total RNA was isolated using the GenElute Mammalian Total RNA Miniprep Kit (Sigma-Aldrich). Reverse transcription was performed using the qScript™ cDNA Synthesis Kit (Quanta Biosciences, Gaithersburg, MD, USA). Both kits were used according to the manufacturer’s manuals. RNA concentrations were measured using the NANODROP 2000 (Thermo Fisher Scientific).

1.10. ChIP and RNA Sequencing, Expression Microarray Hybridization, and Bioinformatics Analyses

ChIP-seq analysis was performed as previously described [3] with minor modifications. Briefly, reads were aligned to hg19 using Bowtie v.1.0.0 allowing a maximum of 2 mismatches and suppressing multiple reads alignments if found. Peak calling was performed using MACS (v macs14 1.4.2 [4])with default parameters and using option “−g 2.63e9” for effective genome size. Reads distribution with bwtool [5] with average of both replicates, motif analysis with de novo discovery settings looking at motifs from 6 bp to 18bp and peaks annotations on genomic features with HOMER package [6].

High confidence maps of genomic regions enriched for H3K27Ac (36357 sites), H3K27me3 (49821 sites), and open chromatin (11065 sites) were compiled with Bedtools [7] intersecting ChIP-seq data for H3K27Ac, H3K27me3 and Formaldehyde assisted isolation of regulatory elements (FAIRE)-seq datasets of 12 melanoma cell lines (MM001, MM011, MM031, MM034, MM047, MM057, MM074, MM087, MM099, MM117, MM118, and SKMEL5) from Verfaillie et al [8]. A separate map was generated intersecting consensus H3K27Ac sites with consensus open chromatin sites (5170 sites). Two maps of high confidence MITF binding sites (MITFBSs) in primary transformed and melanoma cell lines were retrieved by intersecting MITF-BS in transformed melanocytes with enforced expression of BRAFV600E and melanoma cell lines COLO827 (treated with DMSO condition only) from Webster et al [9]. This MITF-BS had 4023 sites. A second high confidence MITF-BS map (234 sites) was build intersecting MITF-BS retrieved in the melanoma cell lines MM011 and MM031 from Verfaillie et al [8].

RNA-seq data on MITF knockdown in 3C and 4C cells were analyzed using STAR (v 20201) with default parameters for alignment to hg19. Resulting bam files were then fed into cuffdiff (v 2.2.1) using all conditions to build base for transcript analysis. A gene was considered to be differentially expressed within a given sample if the following condition was matched: |log_2_ fold change| > 1.

RNA for microaaray expression analysis was hybridized to Illumina (San Diego, CA, USA) platform array according to the manufacturer instruction at the local core facility. The raw signal was then quantile normalized and log _2_-trransformed. A gene was considered to be differentially expressed within a given sample if the following condition was matched: |log_2_ fold change| > 1.5.

Gene ontology and pathway analysis was performed using, geneMANIA [10], MetaScape [11], and GSEA [12]. For each given gene list, pathway and process enrichment analysis has been carried out with the following ontology sources: KEGG Pathway, GO Biological Processes, Reactome Gene Sets, Canonical Pathways and CORUM. For MetaScape and GSEA[12,13] all results are p-adjusted. GeneMANIAwas used with default parameters at genemania.org.

All the data is deposited in GEO under the accession number GSE142018.

1.11. Mutation Analysis of Hermes 3C and 4C Lines Derivatives

Genomic DNA from Hermes cells derivatives was extracted with a Maxwell 16 DNA purification kit (Promega, Madison, WI, USA). Library preparation was carried out using SureSelectXT Human All Exon V5 (Agilent, Santa Clara, CA, USA) per manufacturer’s instructions at the Oslo University Hospital Genomics Core Facility, and whole exome capture and paired-end sequencing was performed using the Illumina HiSeq 2500 platform at an average sequencing depth of 224X. A bioinformatics workflow following GATK best-practices was applied to preprocess the raw sequencing reads, as previously described [14]. Single nucleotide variants and short insertions/deletions were identified with SuperFreq and MuTect2 [15,16]. Variants with a sequencing depth lower than 20 were considered low-confident and discarded. We furthermore used the Personal Cancer Genome Reporter (PCGR v8.0) for functional variant annotation [17]. We applied several filtering procedures available in PCGR to exclude the bulk of known germline variation in the callsets. Specifically, from the initial raw call-set pr. cell line, we excluded: (1) All variants that overlapped germline variants called by the 1000 Genomes Project phase 3 (minor allele frequency (MAF) > 0.2% in any population), or gnomAD (MAF > 0.2% in any population). The MAF threshold (0.2%) was chosen based on recommendations in a recent study on tumor variant filtration strategies [18]. (2) Variants that overlapped any of ~730,000 germline variants detected in an in-house dataset comprising 789 whole exome sequencing samples (blood). (3) Likely heterozygous germline variants, i.e. variants with an allelic fraction between 0.4 and 0.6, and with an existing record in dbSNP or gnomAD. (4) Variants registered in ClinVar with a germline origin. (5) Non-exonic variants (exonic variants retained included coding region variants, i.e. synonymous, missense, stopgain, stoploss, inframe, frameshift, as well as canonical splice site variants (+/− 2bp donor/acceptor)).


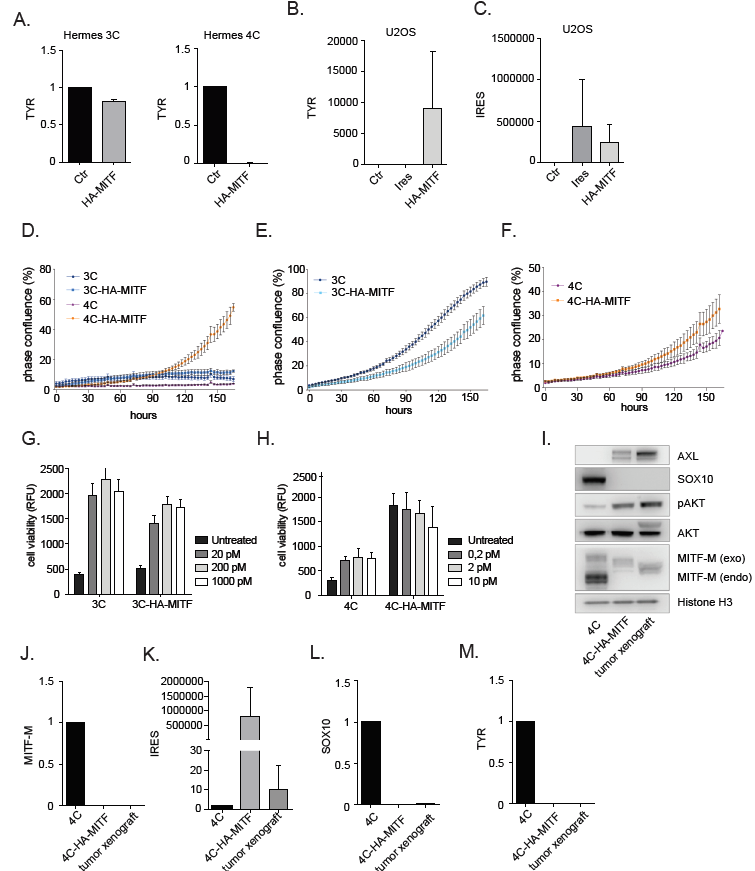


**Figure S1.** HA-MITF-M leads to transformation of immortalized MC1R mutant 4C melanocytes. (**A**) qRT-PCR analysis showing TYR levels in 4C-HA-MITF and 3C-HA-MITF compared to parental 3C and 4C cell lines (**B**) and in U2OS cells transduced with the same HA-MITF construct or with the construct only containing the IRES sequence (control construct) compared to parental U2OS. (**C**) qRT-PCR analysis showing IRES construct levels in U2OS cells transduced with HA-MITF-M construct, or control construct, compared to parental U2OS (*n* = 3). (**D**) Image-based analysis over time of area confluence for the indicated cell lines grown in cholera toxin starved condition. (**E**) Image-based analysis over time of area confluence for the indicated Hermes 3C cell lines derivatives grown in presence of the recommended concentration of cholera toxin (20pM). (**F**) Image-based analysis over time of area confluence for the indicated Hermes 4C cell lines derivatives grown in presence of the recommended concentration of cholera toxin (2 pM). (**G**) Cell proliferation assay (MTS) of Hermes 3C cell lines derivatives grown in presence of the indicated concentrations of cholera toxin for 150 hours. (**H**) Cell proliferation assay (MTS) of Hermes 4C cell lines derivatives grown in presence of the indicated concentrations of cholera toxin for 150 hours. (**I**) Western blot analysis of endogenous (endo) and ectopically transduced (exo) MITF-M in Hermes 4C parental cells, 4C-HA-MITF, and a representative tumor xenograft. Expression analysis of SOX10, AXL, pAKT and total AKT are also shown. The image is representative of n = 3 experiments. qRT-PCR gene expression analyses of (endogenous) MITF-M (**J**) IRES construct (**K**), SOX10 (**L**), and TYR (**M**) transcripts in 4C, 4C-HA-MITF, and a representative tumor xenograft compared to expression levels in parental 4C cells. Histone H3 was used as loading control. Representative blot from three different experiments.

**Figure S2.** HA-MITF directs specific transcription leading to melanocyte transformation. **(A)** Western blot analyses of MITF-M in the indicated Hermes parental lines untransfected (Ctr), or upon transfection with siRNA control (Scr), or siRNA directed against MITF-M. Gene Ontology, pathway and function analysis of **(B)** genes downregulated upon MITF-M knockdown in Hermes 3C cells and **(C)** genes upregulated upon MITF-M knockdown in Hermes 3C cells, **(D)** genes upregulated upon MITF-M knockdown in Hermes 4C cells and **(E)** genes downregulated upon MITF-M knockdown in Hermes 4C cells (analysis of GO and pathway analysis with metascape is also shown in the left bottom panel). **(F)** Metascape Gene Ontology (GO), pathway and function analysis of genes upregulated upon MITF-M knockdown in exclusively in Hermes 4C cells but not in Hermes 3C cells. Clustering analysis of Hermes cells derivatives transduced with HA-MITF or control vector according to the expression of 200 genes included in the epithelial to mesenchymal transition (EMT) hallmark signature **(G)** and according to the expression of genes included in the core signature of the invasive melanoma phenotype **(H)** by Verfaille et al. [8].


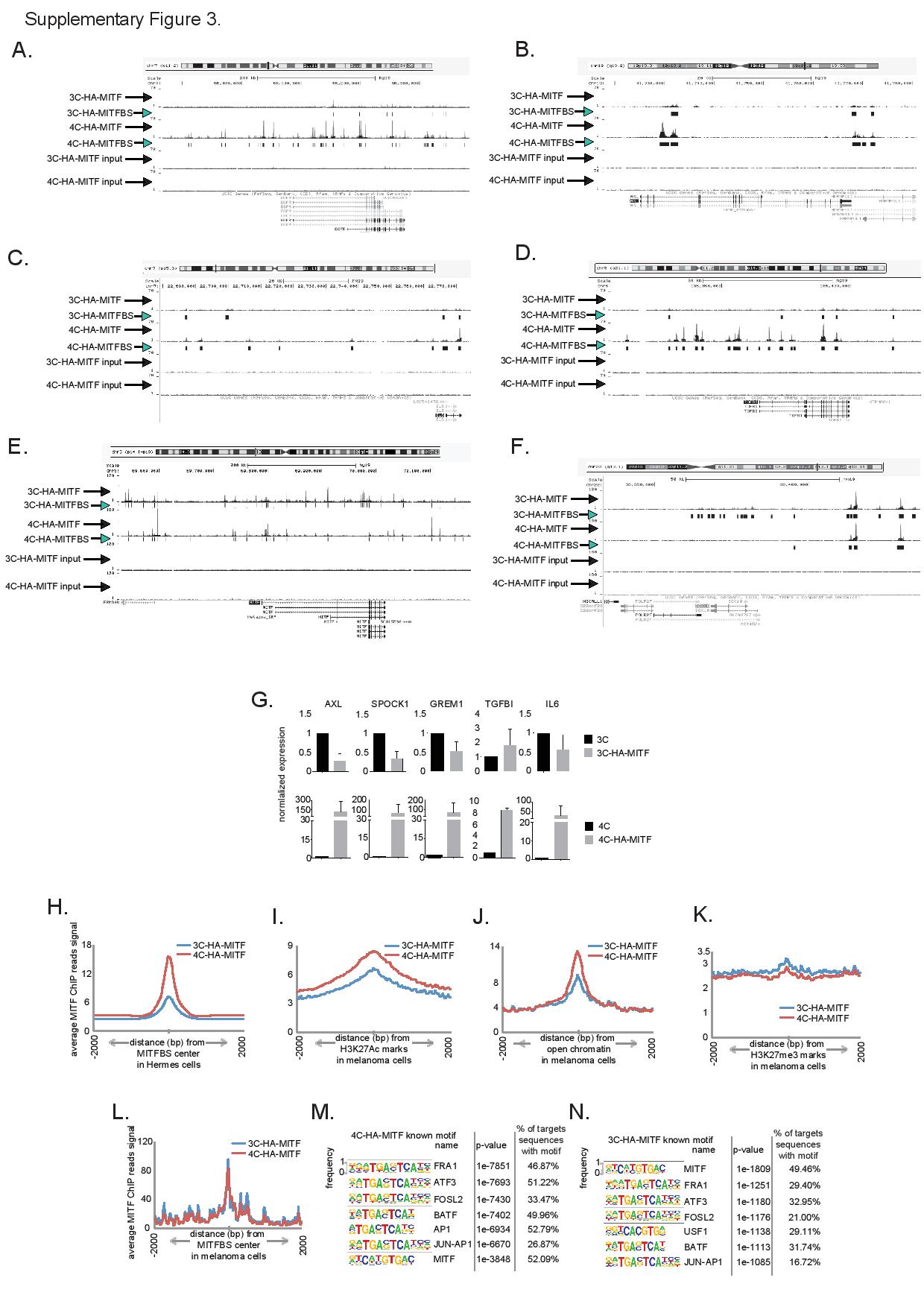


**Figure S3.** HA-MITF-M enhances the chromatin binding. Representative UCSC genome browser snapshot showing enrichment of MITF-M binding in proximity of EGFR (**A**), AXL gene (**B**), the IL6 gene (**C**), the TGFBI gene (**D**), MITF (**E**), and SOX10 (**F**) genes. The UCSC genome browser normalized bigwig files tracks are labeled and indicated by the black horizontal arrow on the left side of each panel. Shorter green horizontal arrows point at BED files tracks consensus MITF binding sites (MITFBS) in each cell lines. (**G**) Expression analysis of the indicated MITF target genes using RT-PCR in Hermes cell derivatives. Graphs represents expression data from three separate experiments normalized to control and plotted as mean±SD. (**H**) Distribution analysis of normalized MITF ChIP-seq reads in 3C-HA-MITF and 4C-HAMITF cells around the compendium of all MITF binding sites (MITFBSs) in Hermes cells. (**I**) Distribution analysis of normalized MITF ChIP-seq reads in 3C-HA-MITF and 4C-HA-MIT cells around high confidence H3K27Ac marked sites in 12 melanoma cell lines from Verfaillie et al (6) (see Methods). (**J**) Distribution analysis of normalized MITF ChIP-seq reads in 3C-HA-MITF and 4C-HA-MITF cells around high confidence open chromatin in 12 melanoma cell lines from Verfaillie et al (6) (see Methods). (**K**) Distribution analysis of normalized MITF ChIP-seq reads in 3C-HA-MITF and 4C-HA-MITF cells around high confidence H3K27me3 marked sites in 12 melanoma cell lines from Verfaillie et al (6) (see Methods). (**L**) Distribution analysis of normalized MITF ChIP-seq reads in 3C-HA-MITF and 4C-HA-MITF cells around high confidence MITFBSs in the melanoma cell lines MM011 and MM931 from Verfaillie et al (6) (see Methods). Note that all these sites were present in the sites relative to main figure 2E. (**M**) Known DNA motifs enrichment analysis using 41200 MITFBSs’ DNA sequences retrieved from 3C-HA-MITF cells. (**N**) Known DNA motifs enrichment analysis using 80070MITFBSs’ DNA sequences retrieved from 4C-HA-MITF cells.


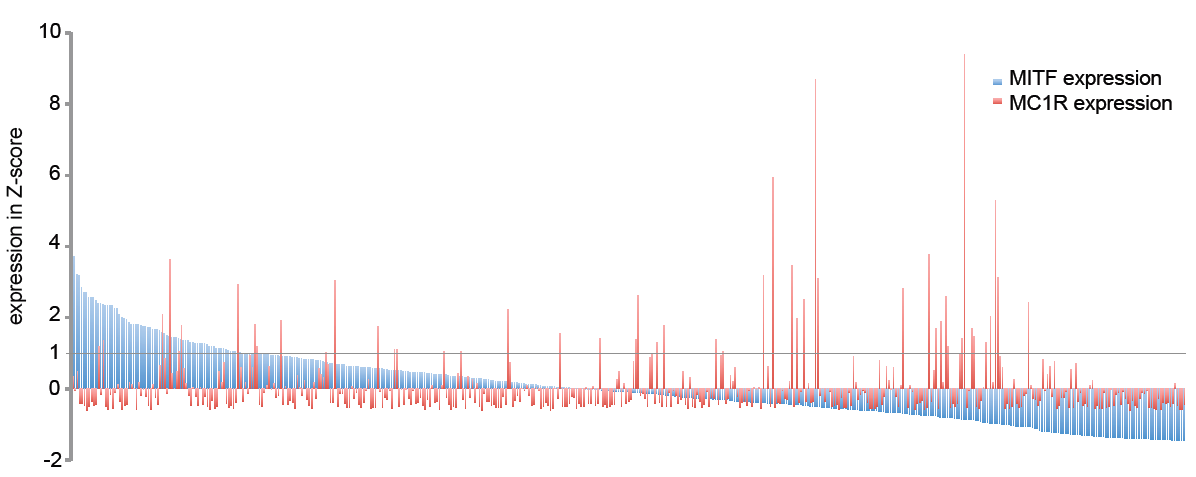


**Figure S4.** MITF-M and MC1R in the TCGA dataset**.** Expression analysis of MC1R and MITF genes in the 471 tumors from the TCGA melanoma cohort.

**
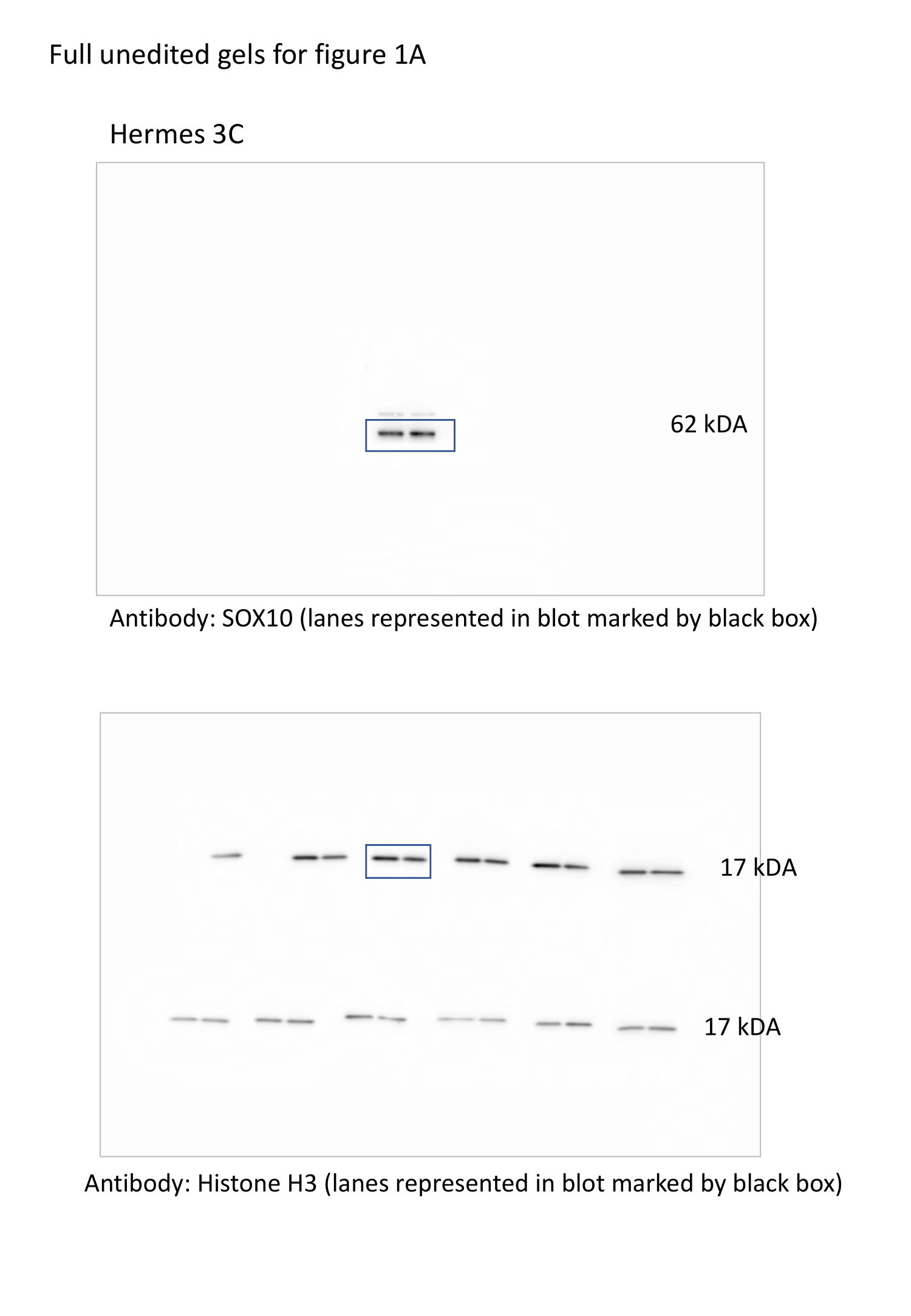
**
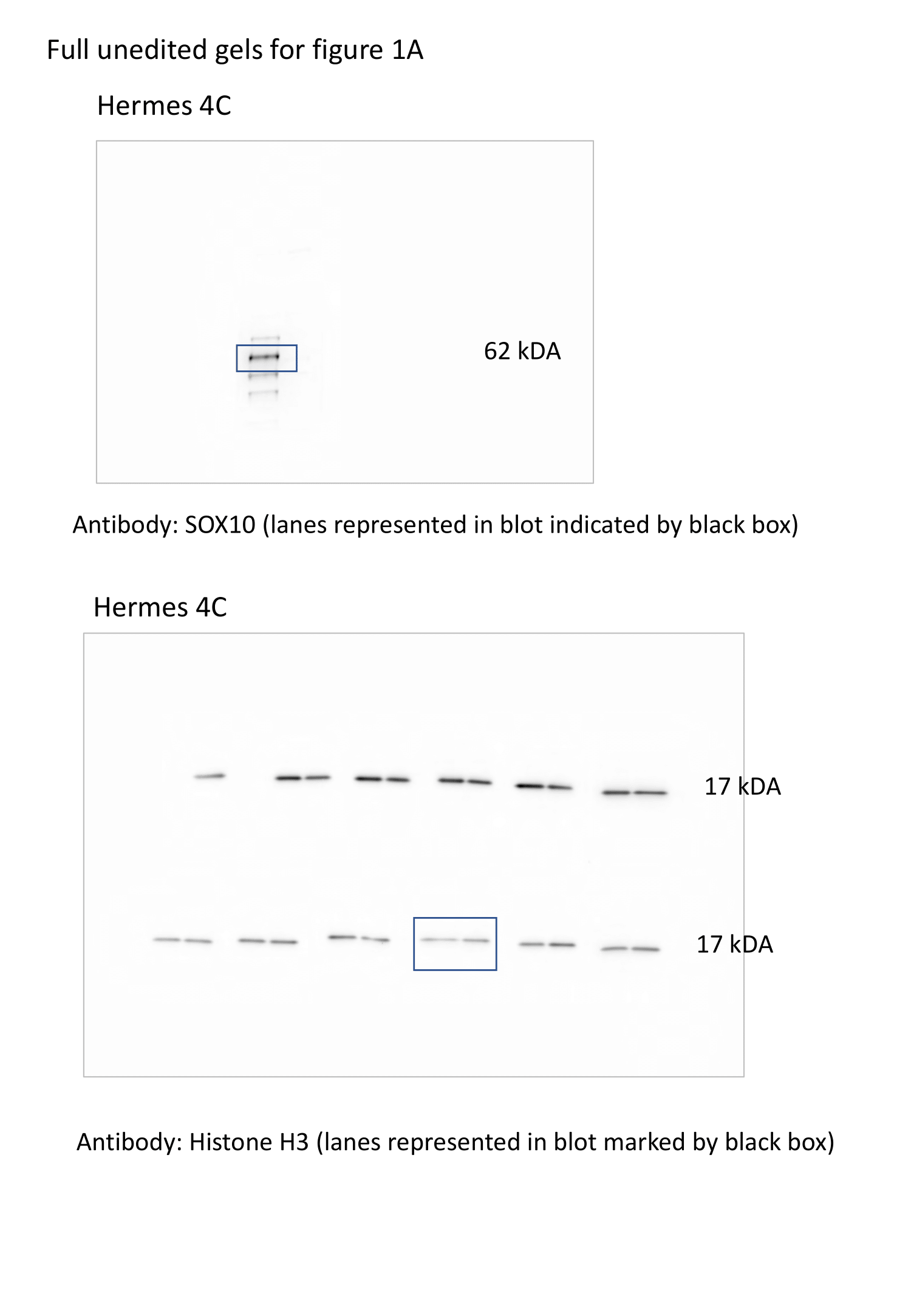


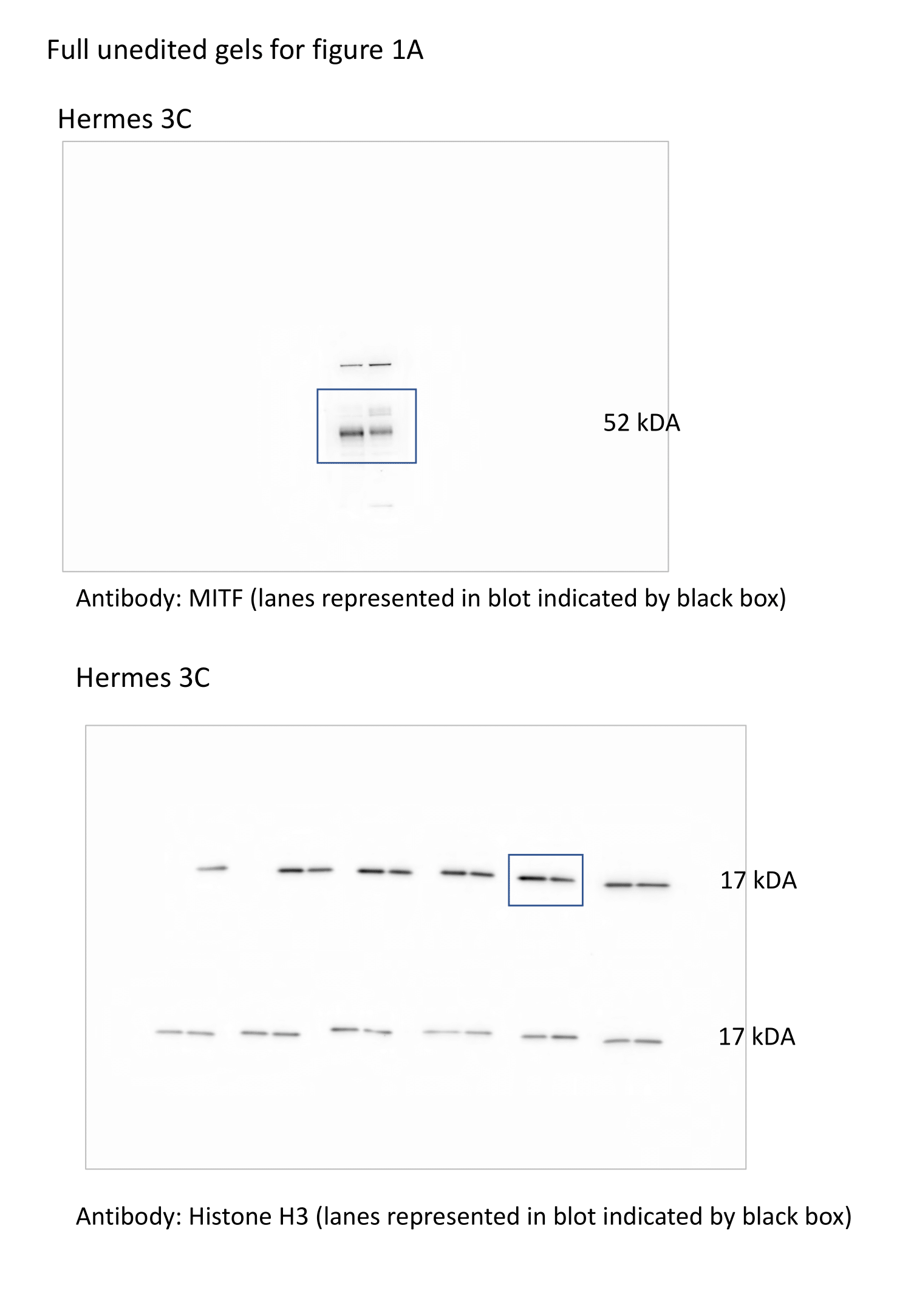


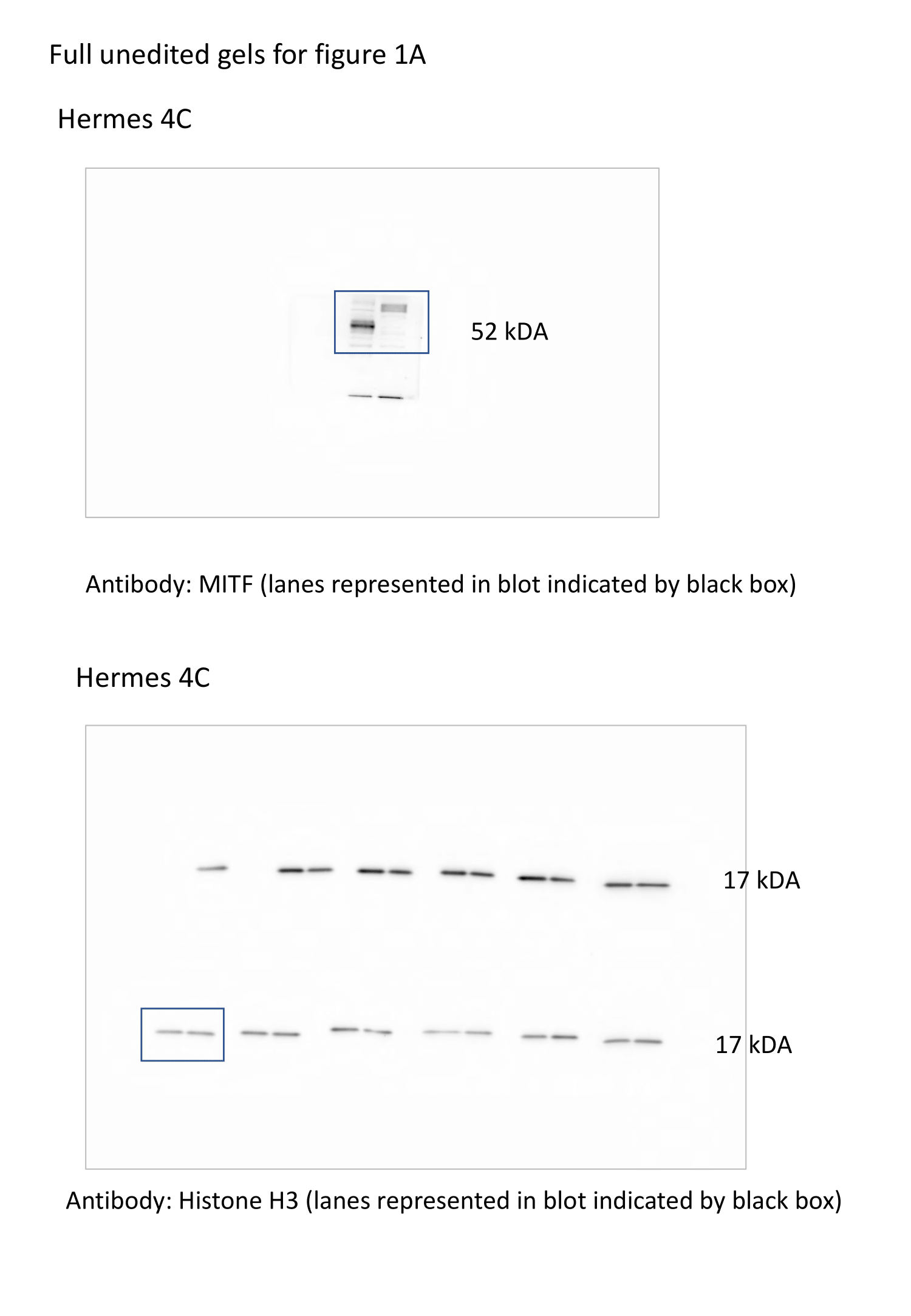


**
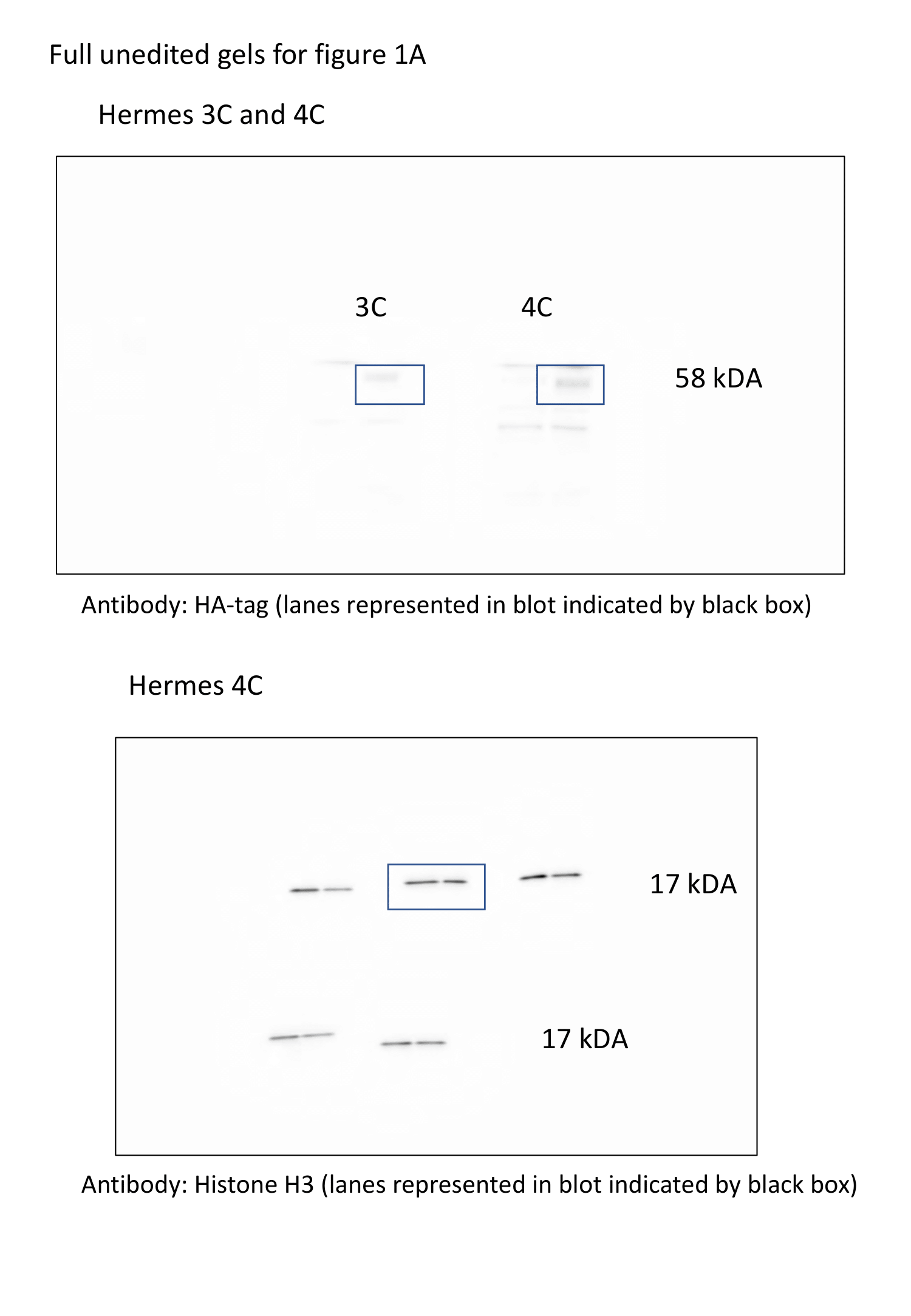
**

**
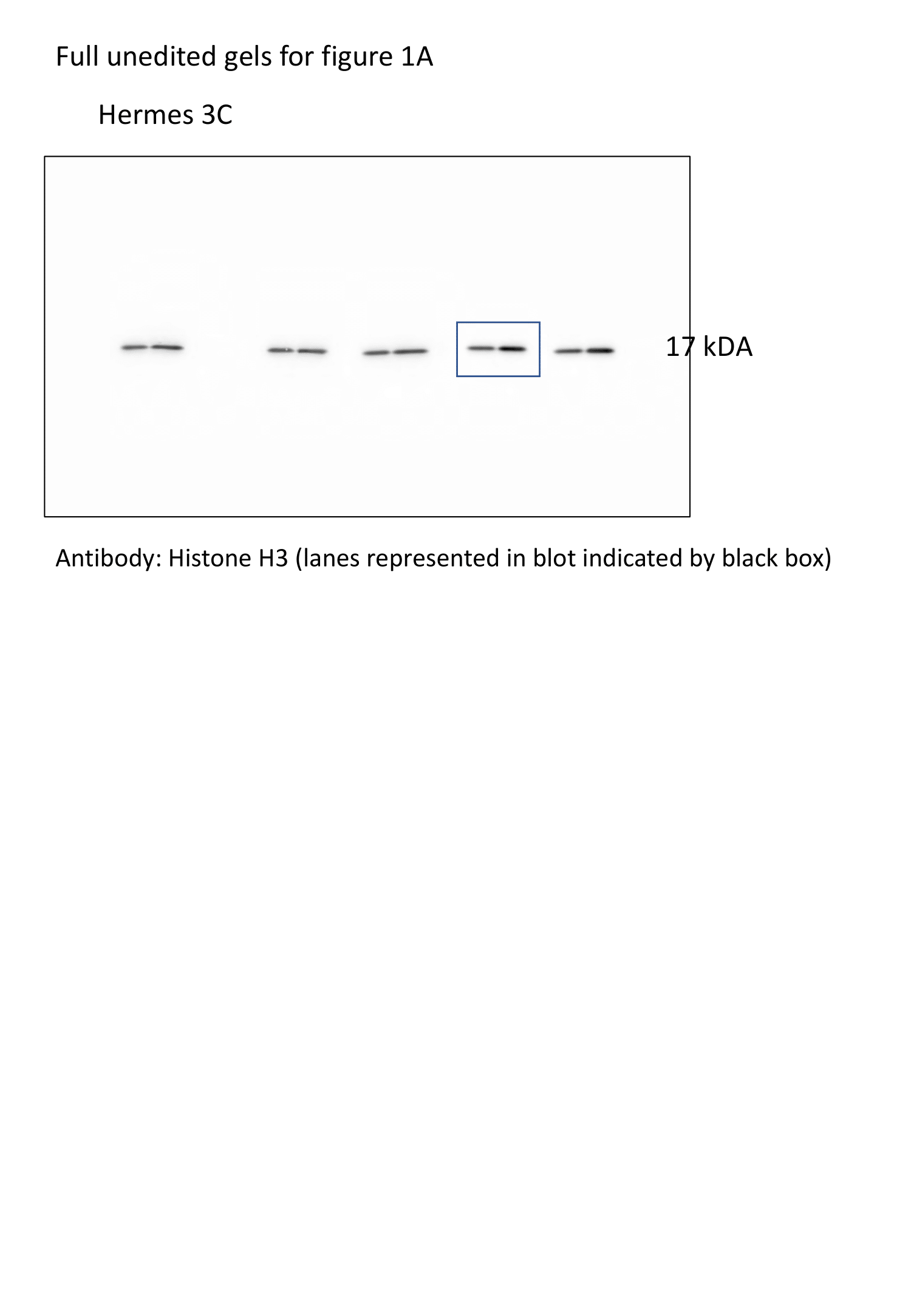
**

**
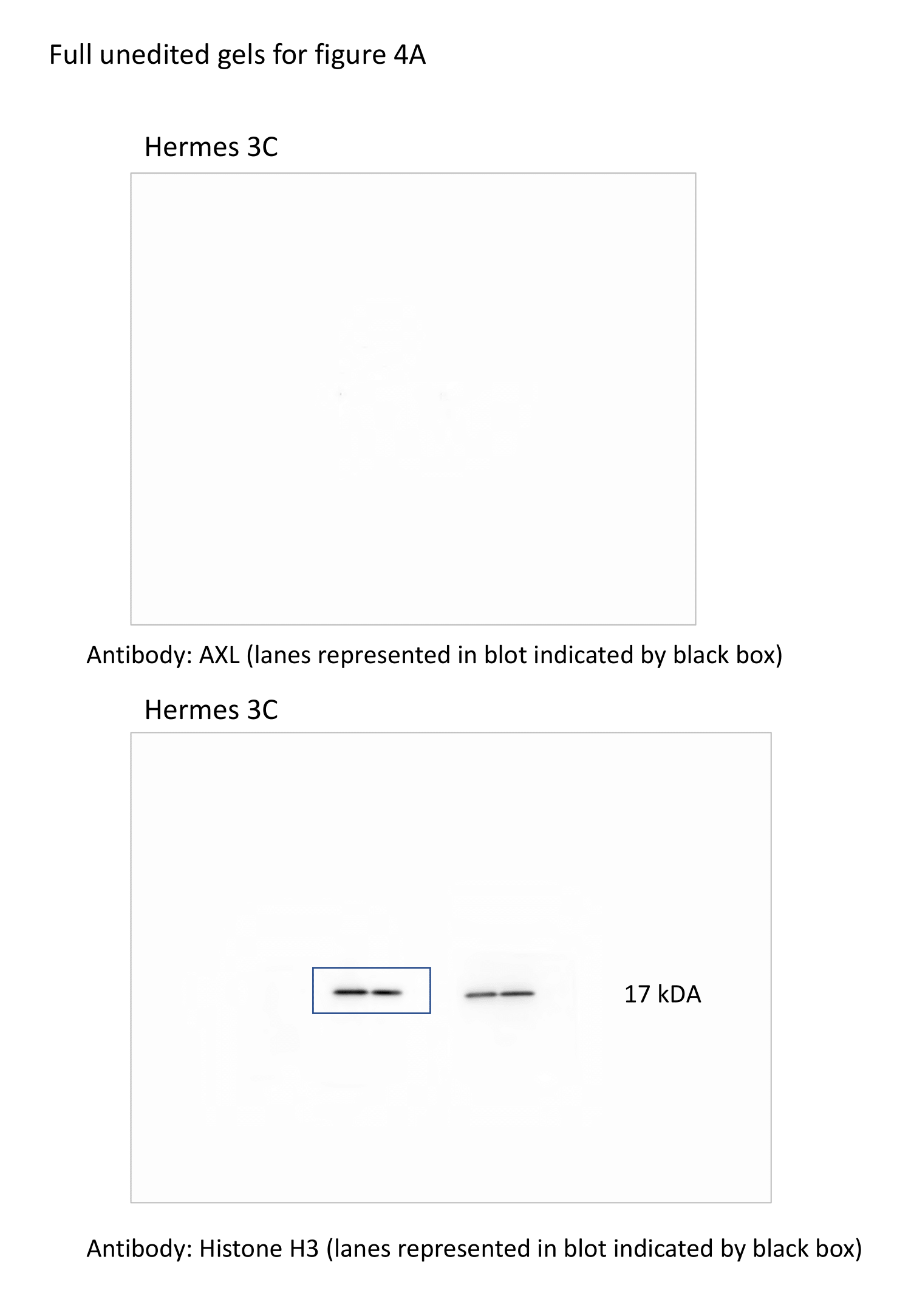
**

**
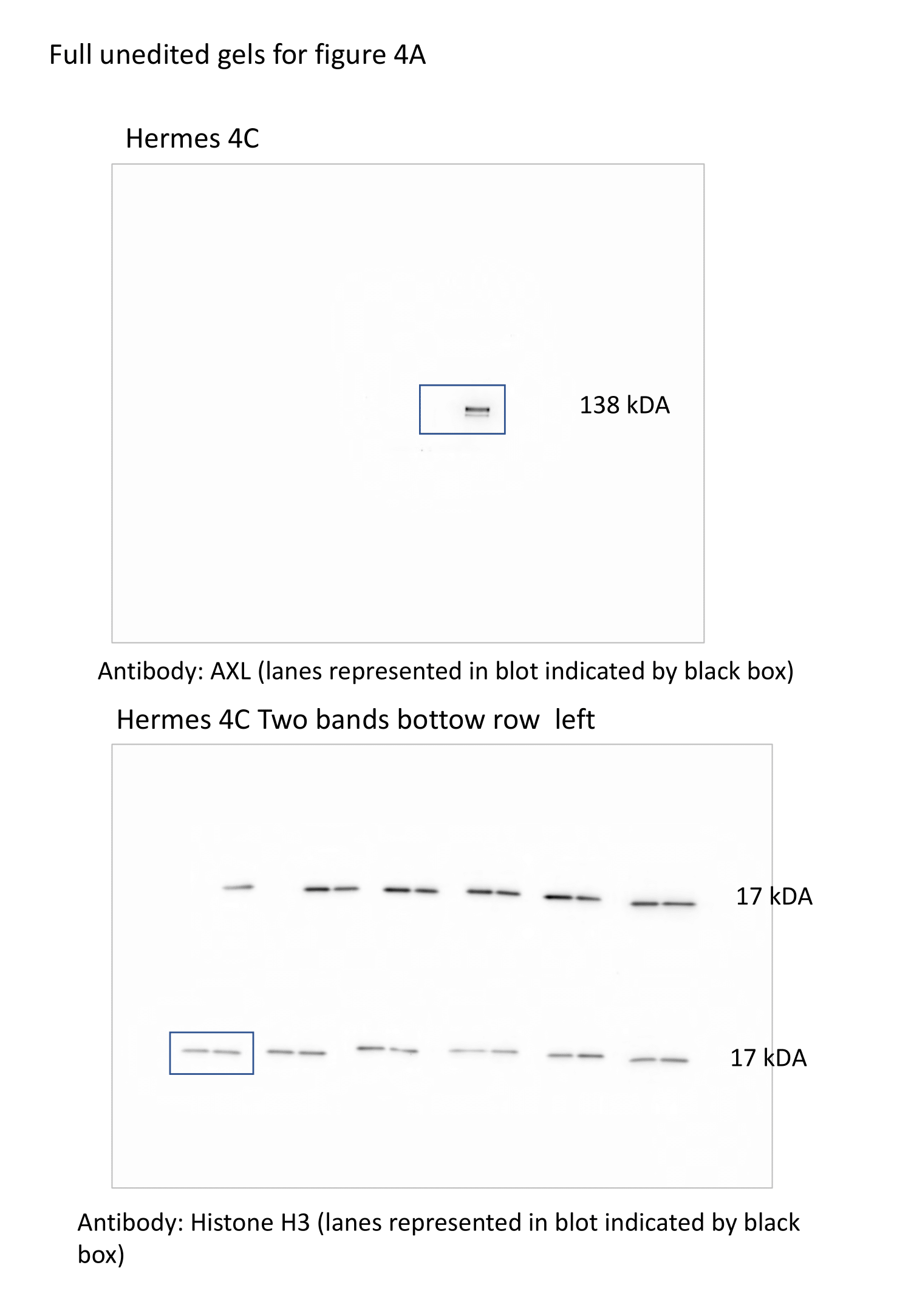
**

**
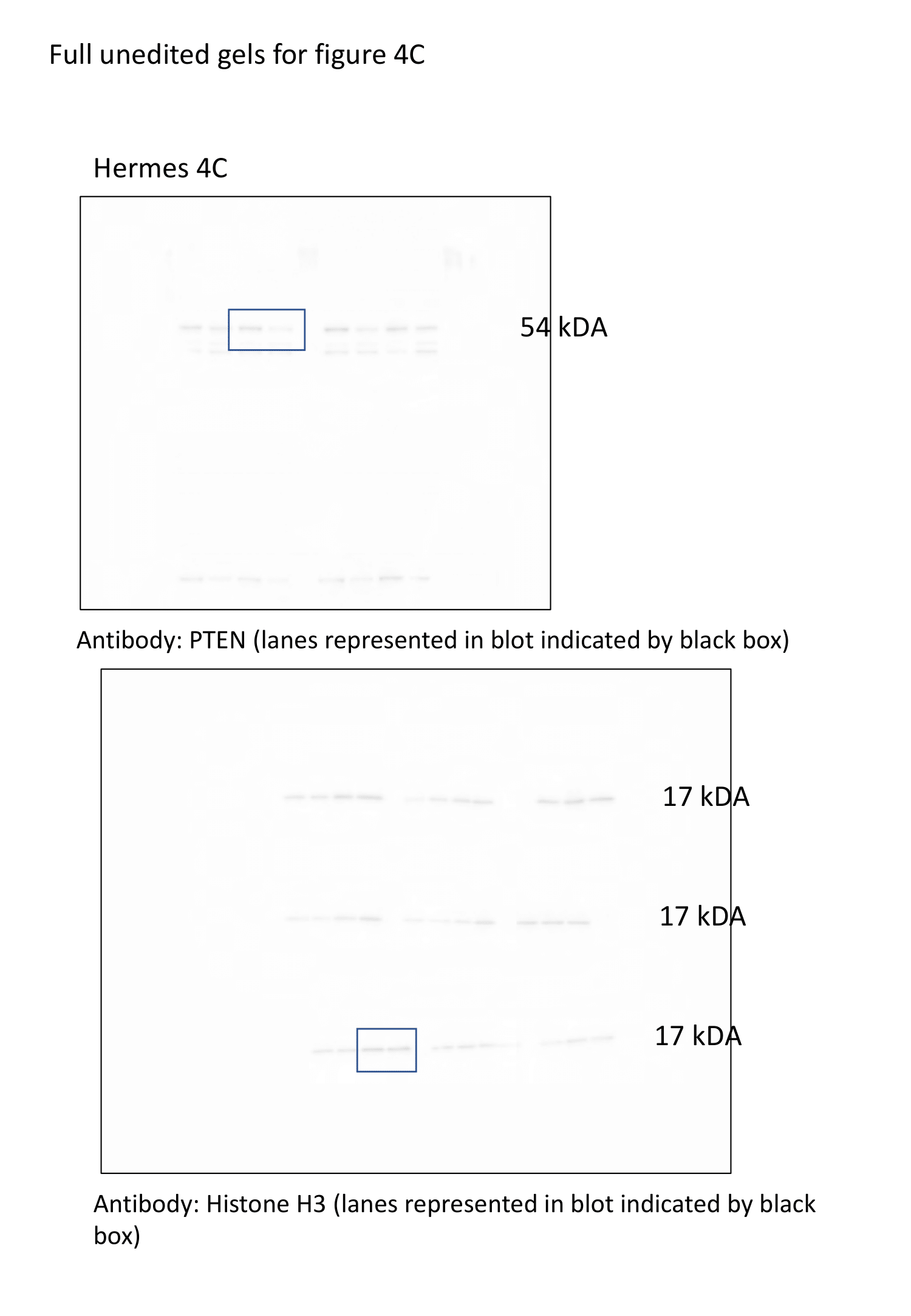
**

**
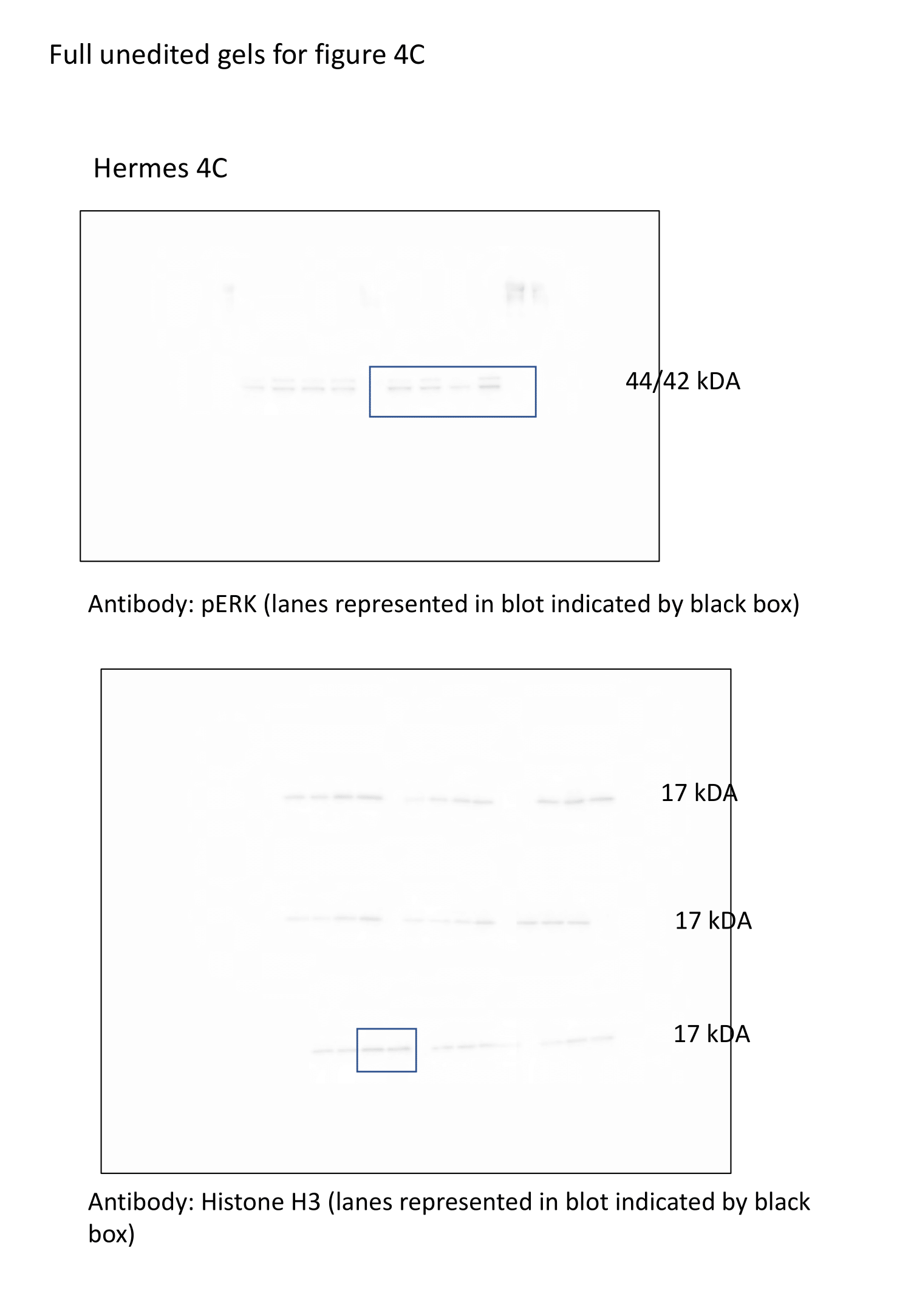
**

**
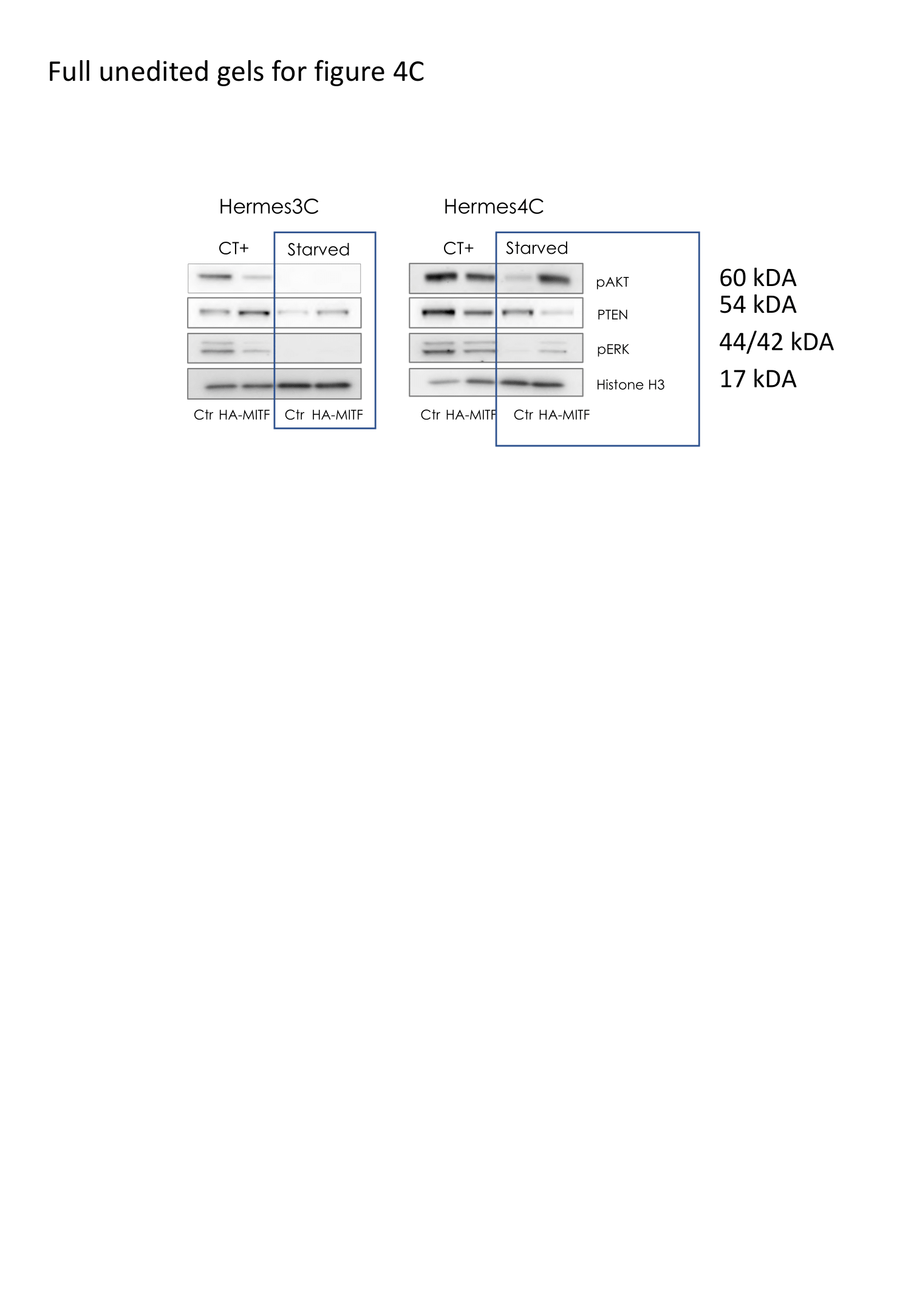
**

**
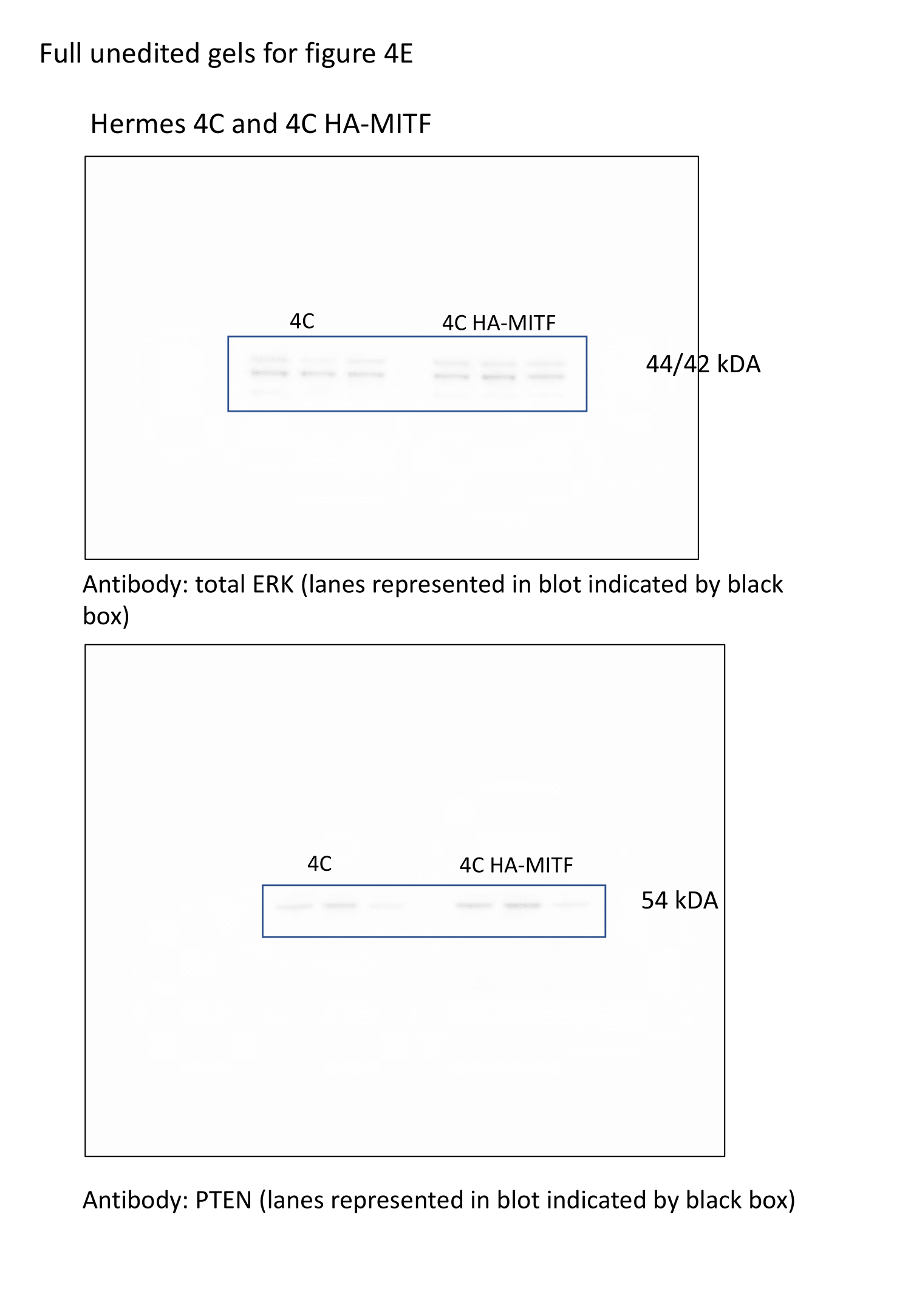
**

**
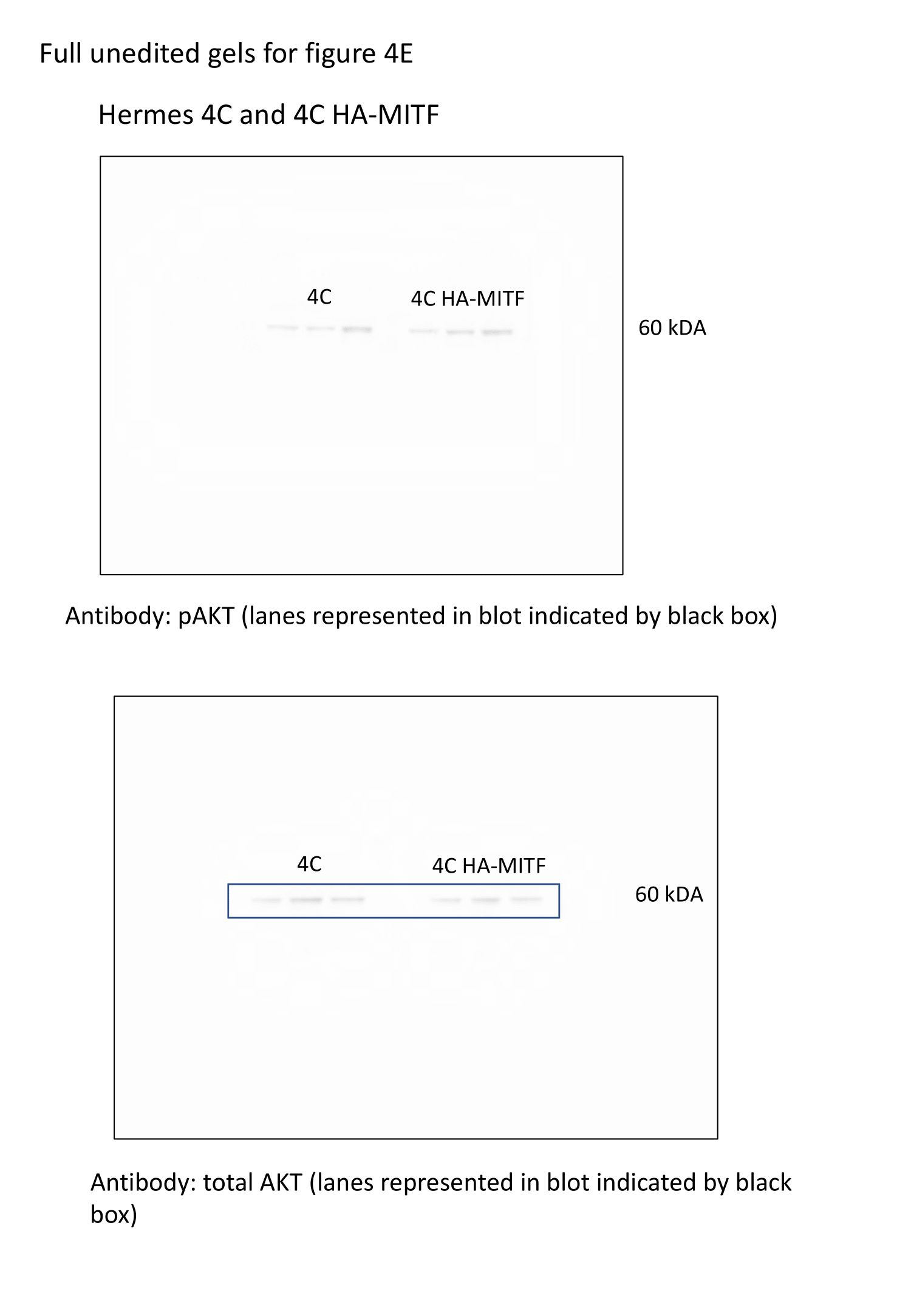
**

**
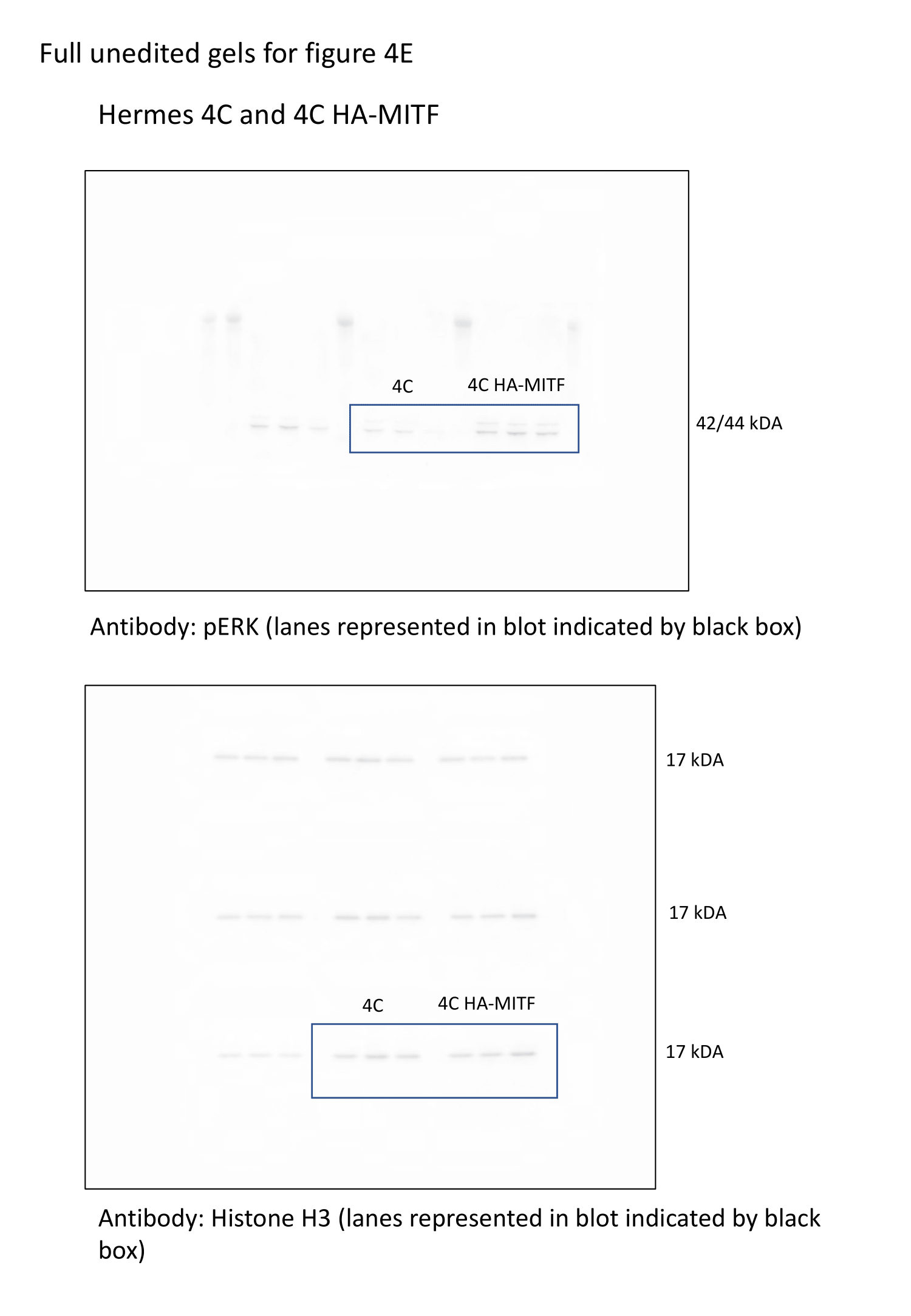
**

**
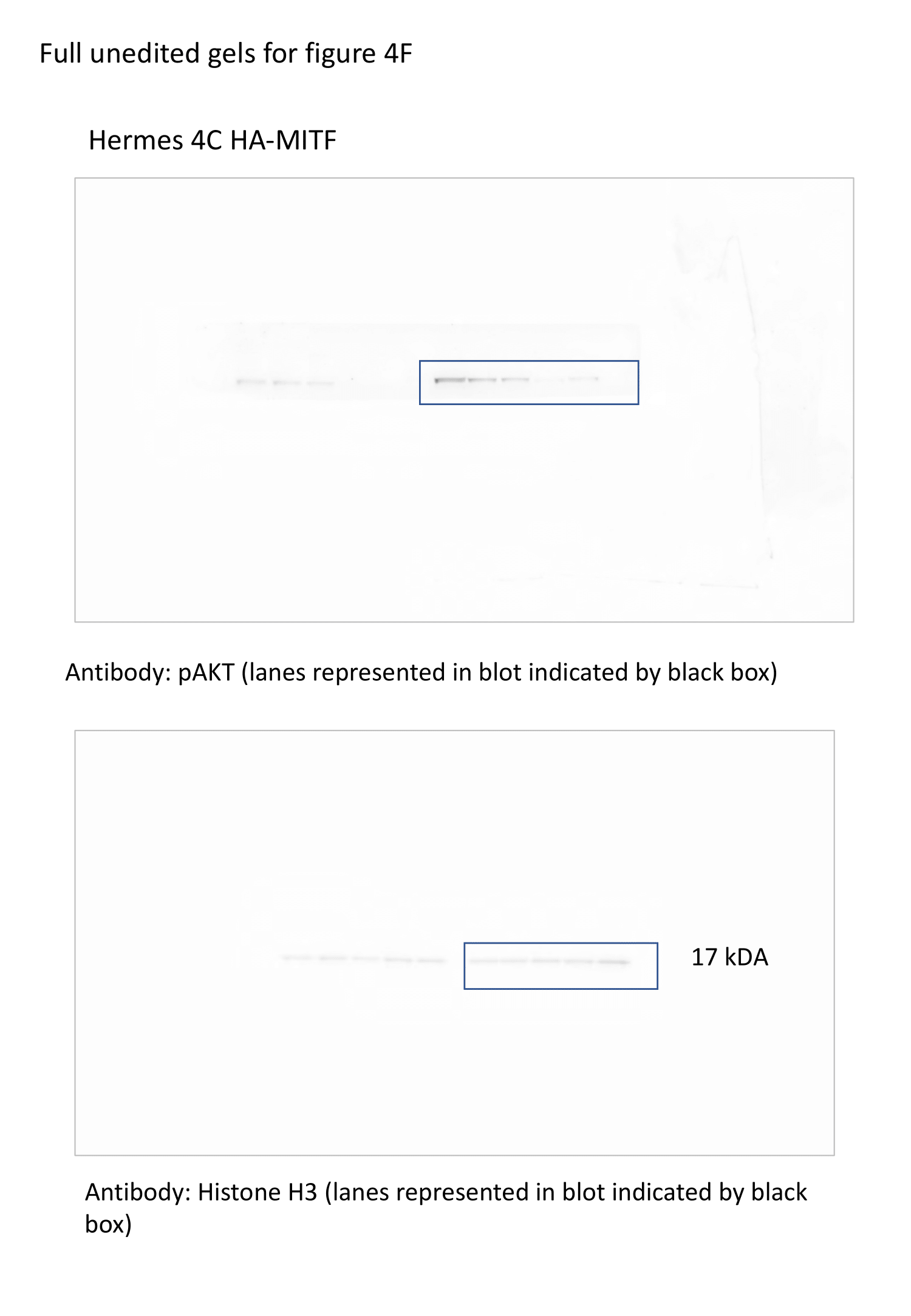
**

**
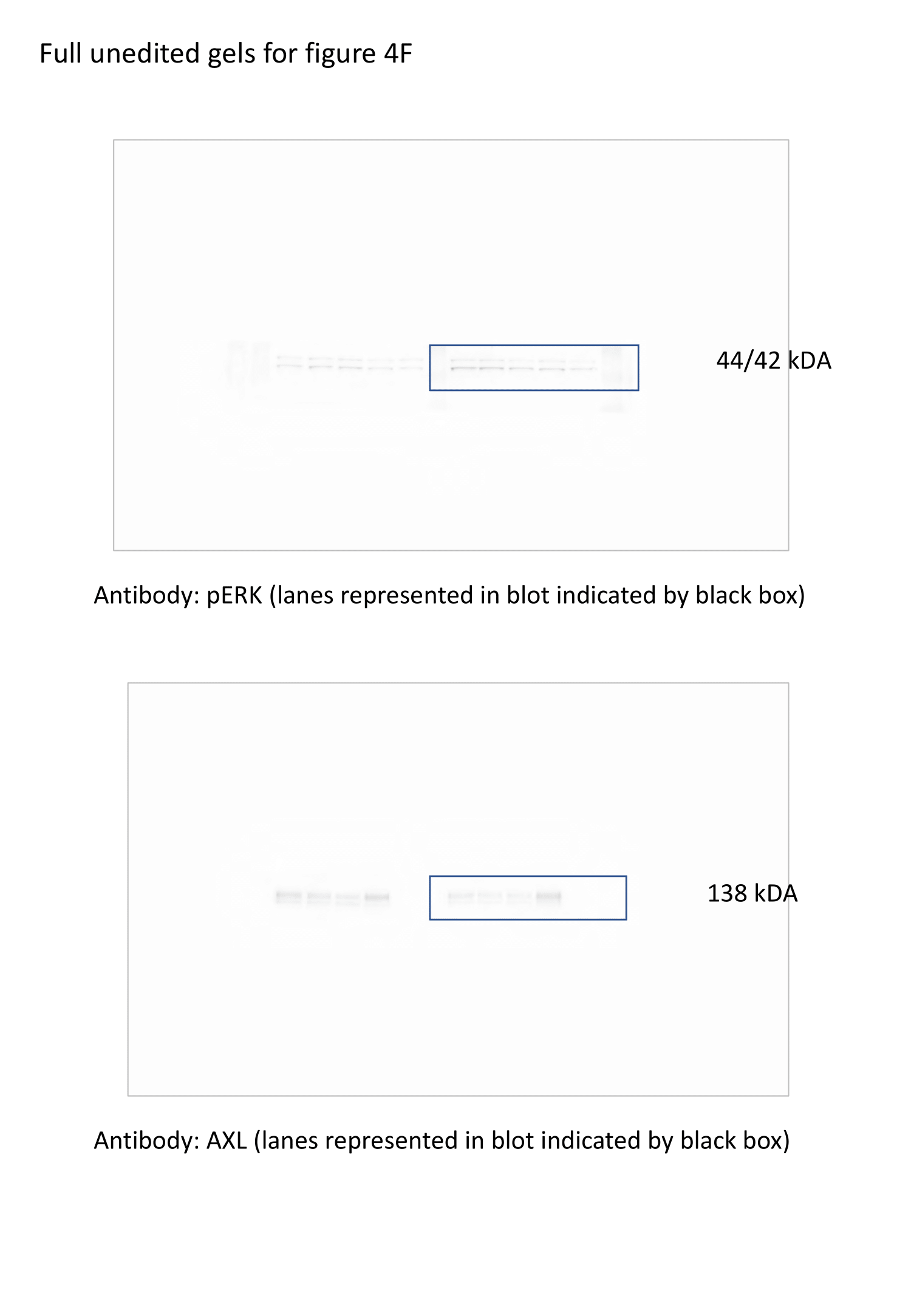
**

**
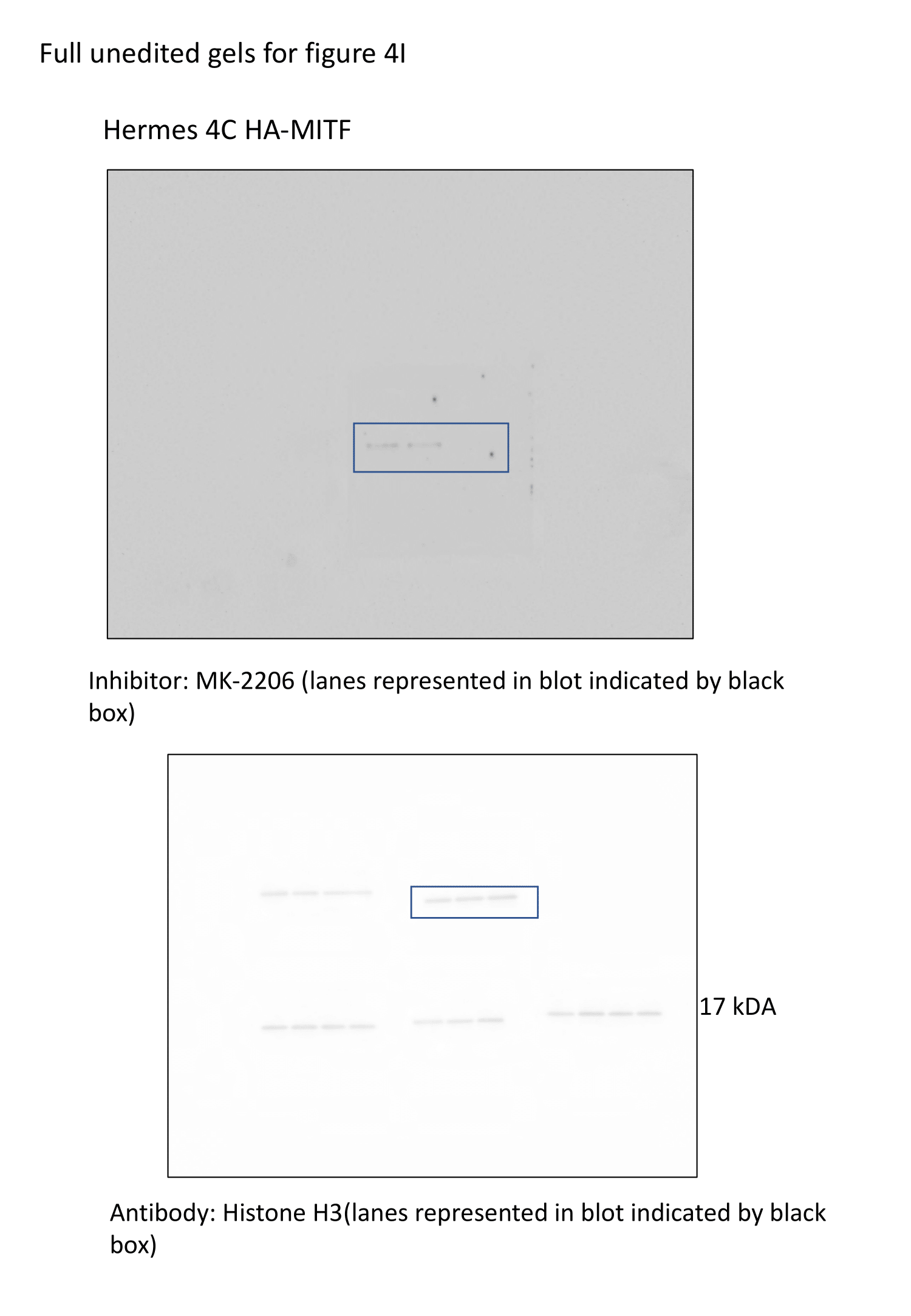
**

**
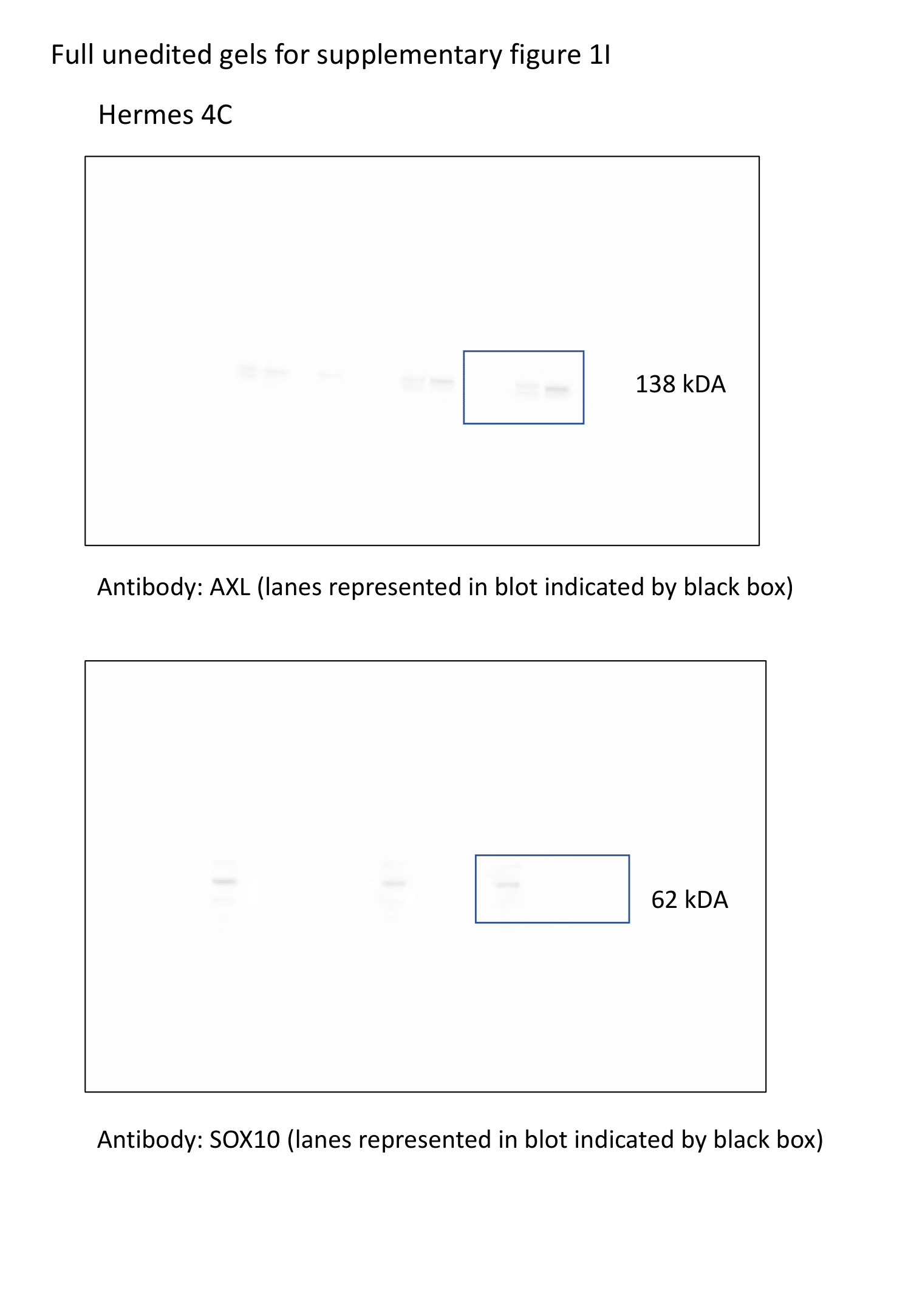
**

**
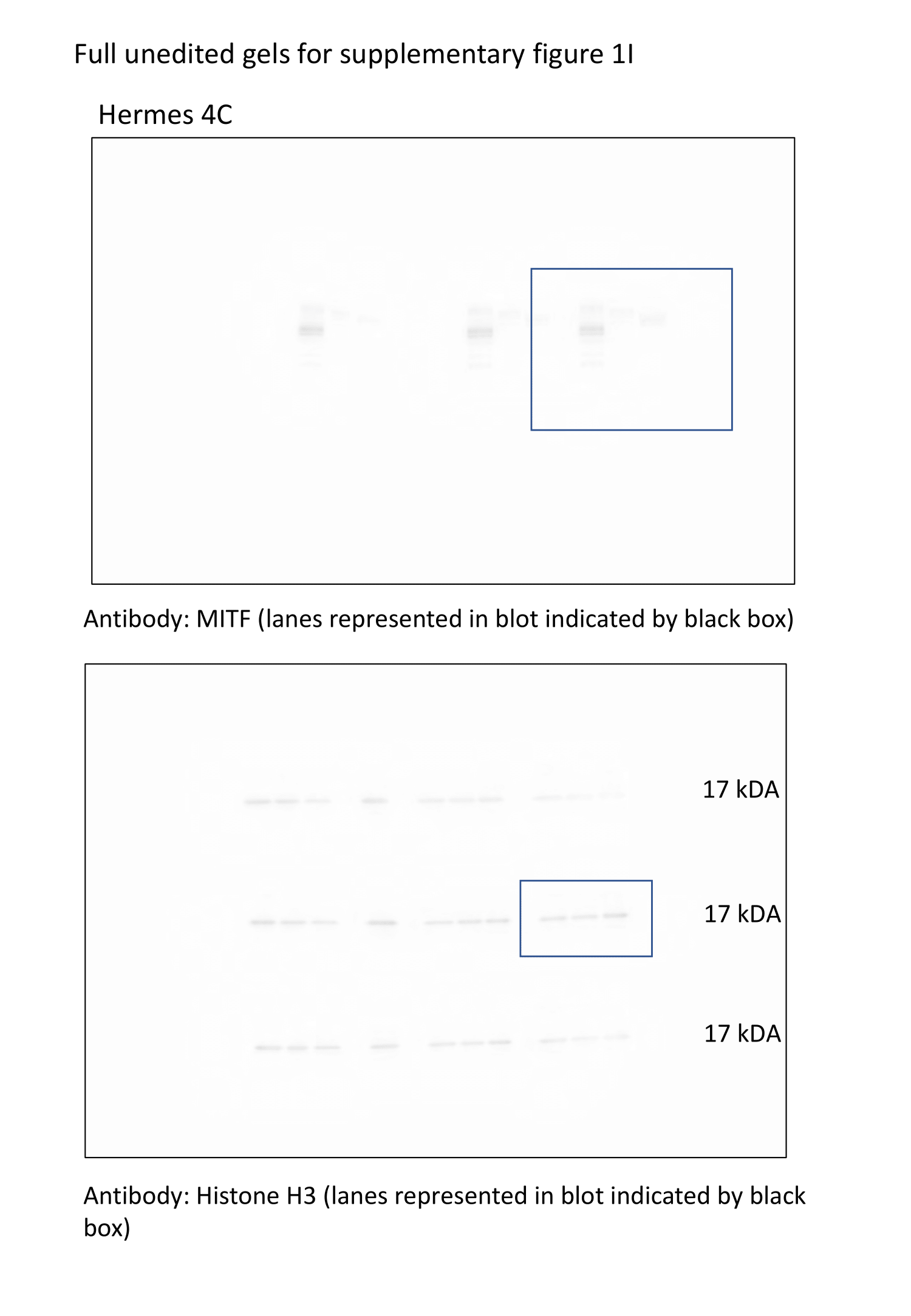
**

**
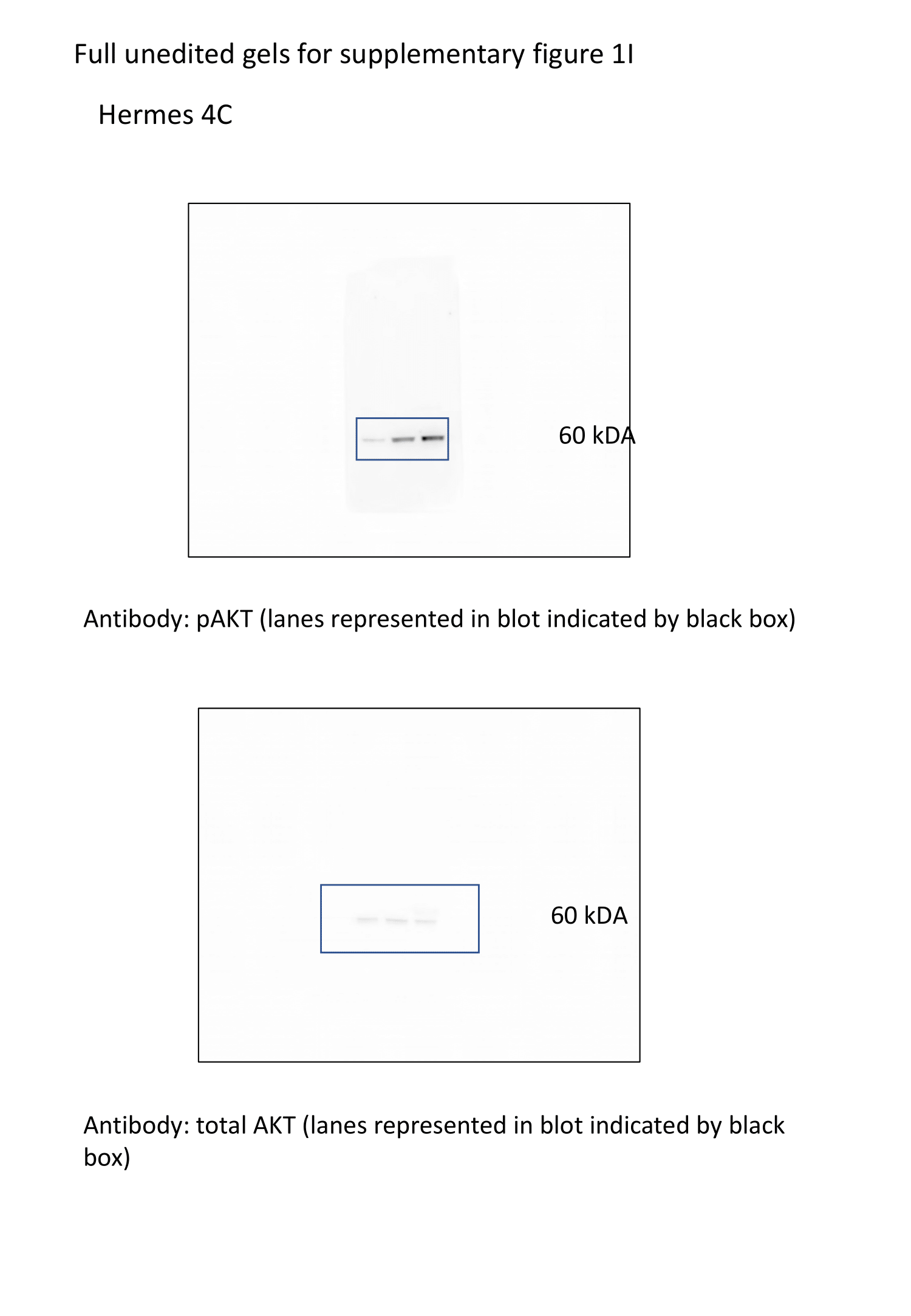
**


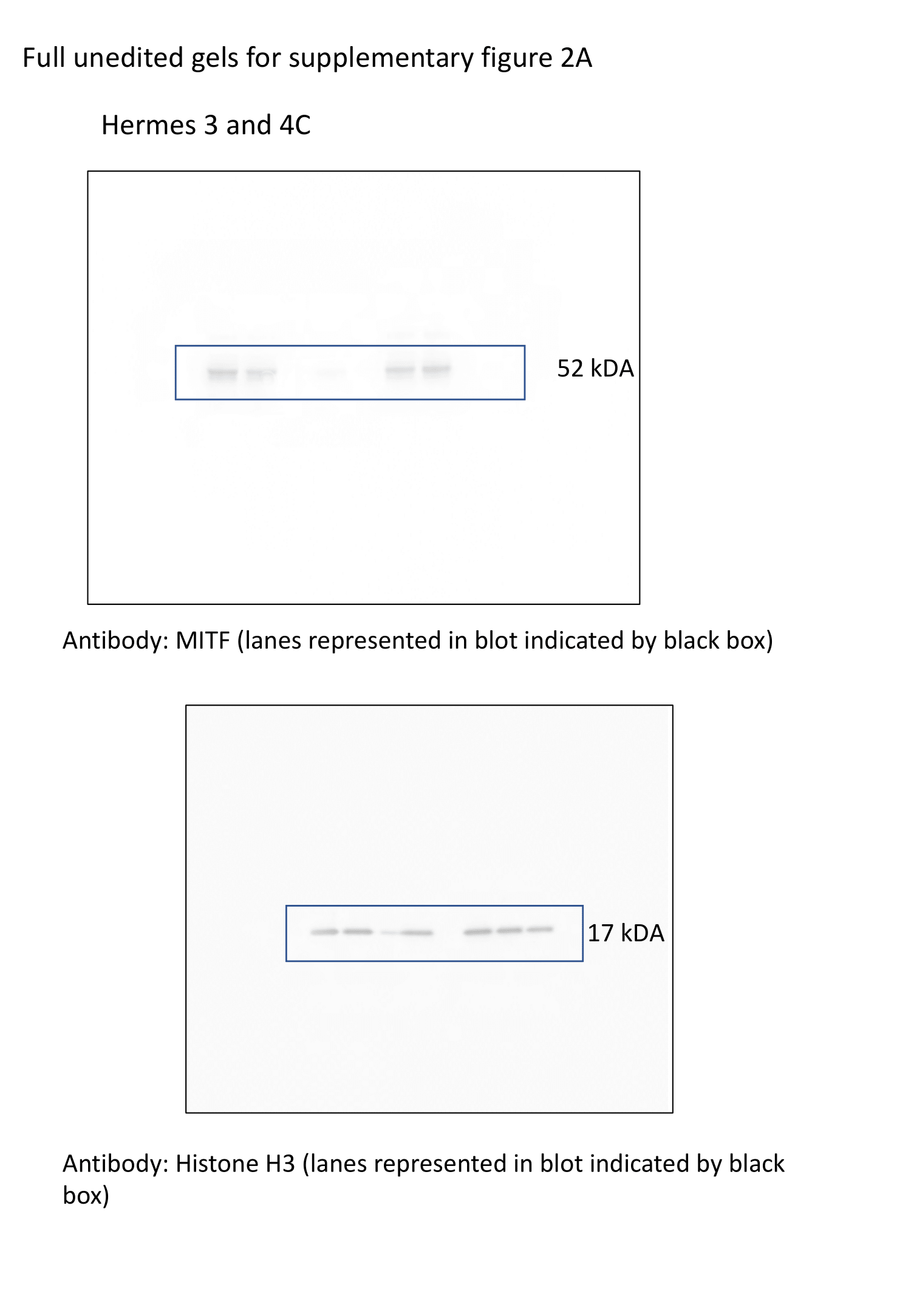


**Figure S5.** Uncropped blots relative to Figures 1A, 4A, 4C, 4E, 4F, and 4I and to Supplementary Figures S1I and S2A.

**References**

1. Strub, T.; Giuliano, S.; Ye, T.; Bonet, C.; Keime, C.; Kobi, D.; Le Gras, S.; Cormont, M.; Ballotti, R.; Bertolotto, C., et al. Essential role of microphthalmia transcription factor for DNA replication, mitosis and genomic stability in melanoma. *Oncogene* **2011**, *30*, 2319–2332, doi:10.1038/onc.2010.612.

2. Stewart, S.A.; Dykxhoorn, D.M.; Palliser, D.; Mizuno, H.; Yu, E.Y.; An, D.S.; Sabatini, D.M.; Chen, I.S.; Hahn, W.C.; Sharp, P.A., et al. Lentivirus-delivered stable gene silencing by RNAi in primary cells. *Rna* **2003**, *9*, 493–501, doi:10.1261/rna.2192803.

3. Urbanucci, A.; Barfeld, S.J.; Kytola, V.; Itkonen, H.M.; Coleman, I.M.; Vodak, D.; Sjoblom, L.; Sheng, X.; Tolonen, T.; Minner, S., et al. Androgen Receptor Deregulation Drives Bromodomain-Mediated Chromatin Alterations in Prostate Cancer. *Cell Rep.* **2017**, *19*, 2045–2059, doi:10.1016/j.celrep.2017.05.049.

4. Zhang, Y.; Liu, T.; Meyer, C.A.; Eeckhoute, J.; Johnson, D.S.; Bernstein, B.E.; Nusbaum, C.; Myers, R.M.; Brown, M.; Li, W., et al. Model-based analysis of ChIP-Seq (MACS). *Genome Biol.* **2008**, *9*, R137, doi:10.1186/gb-2008-9-9-r137.

5. Pohl, A.; Beato, M. bwtool: a tool for bigWig files. *Bioinformatics* **2014**, *30*, 1618–1619, doi:10.1093/bioinformatics/btu056.

6. Heinz, S.; Benner, C.; Spann, N.; Bertolino, E.; Lin, Y.C.; Laslo, P.; Cheng, J.X.; Murre, C.; Singh, H.; Glass, C.K. Simple combinations of lineage-determining transcription factors prime cis-regulatory elements required for macrophage and B cell identities. *Mol. Cell* **2010**, *38*, 576–589, doi:10.1016/j.molcel.2010.05.004.

7. Quinlan, A.R.; Hall, I.M. BEDTools: a flexible suite of utilities for comparing genomic features. *Bioinformatics* **2010**, *26*, 841–842, doi:10.1093/bioinformatics/btq033.

8. Verfaillie, A.; Imrichova, H.; Atak, Z.K.; Dewaele, M.; Rambow, F.; Hulselmans, G.; Christiaens, V.; Svetlichnyy, D.; Luciani, F.; Van den Mooter, L., et al. Decoding the regulatory landscape of melanoma reveals TEADS as regulators of the invasive cell state. *Nat. Commun.* **2015**, *6*, 6683, doi:10.1038/ncomms7683.

9. Webster, D.E.; Barajas, B.; Bussat, R.T.; Yan, K.J.; Neela, P.H.; Flockhart, R.J.; Kovalski, J.; Zehnder, A.; Khavari, P.A. Enhancer-targeted genome editing selectively blocks innate resistance to oncokinase inhibition. *Genome Res.* **2014**, *24*, 751–760, doi:10.1101/gr.166231.113.

10. Warde-Farley, D.; Donaldson, S.L.; Comes, O.; Zuberi, K.; Badrawi, R.; Chao, P.; Franz, M.; Grouios, C.; Kazi, F.; Lopes, C.T., et al. The GeneMANIA prediction server: biological network integration for gene prioritization and predicting gene function. *Nucleic Acids Res.* **2010**, *38*, W214–220, doi:10.1093/nar/gkq537.

11. Zhou, Y.; Zhou, B.; Pache, L.; Chang, M.; Khodabakhshi, A.H.; Tanaseichuk, O.; Benner, C.; Chanda, S.K. Metascape provides a biologist-oriented resource for the analysis of systems-level datasets. *Nat. Commun.* **2019**, *10*, 1523, doi:10.1038/s41467-019-09234-6.

12. Subramanian, A.; Tamayo, P.; Mootha, V.K.; Mukherjee, S.; Ebert, B.L.; Gillette, M.A.; Paulovich, A.; Pomeroy, S.L.; Golub, T.R.; Lander, E.S., et al. Gene set enrichment analysis: a knowledge-based approach for interpreting genome-wide expression profiles. *Proc. Natl. Acad. Sci. USA* **2005**, *102*, 15545–15550, doi:10.1073/pnas.0506580102.

13. Mootha, V.K.; Lindgren, C.M.; Eriksson, K.-F.; Subramanian, A.; Sihag, S.; Lehar, J.; Puigserver, P.; Carlsson, E.; Ridderstråle, M.; Laurila, E., et al. PGC-1α-responsive genes involved in oxidative phosphorylation are coordinately downregulated in human diabetes. *Nat. Genet.* **2003**, *34*, 267–273, doi:10.1038/ng1180.

14. Birkeland, E.; Zhang, S.; Poduval, D.; Geisler, J.; Nakken, S.; Vodak, D.; Meza-Zepeda, L.A.; Hovig, E.; Myklebost, O.; Knappskog, S., et al. Patterns of genomic evolution in advanced melanoma. *Nat. Commun.* **2018**, *9*, 2665, doi:10.1038/s41467-018-05063-1.

15. Cibulskis, K.; Lawrence, M.S.; Carter, S.L.; Sivachenko, A.; Jaffe, D.; Sougnez, C.; Gabriel, S.; Meyerson, M.; Lander, E.S.; Getz, G. Sensitive detection of somatic point mutations in impure and heterogeneous cancer samples. *Nat. Biotechnol.* **2013**, *31*, 213–219, doi:10.1038/nbt.2514.

16. Flensburg, C.; Sargeant, T.; Oshlack, A.; Majewski, I. SuperFreq: Integrated mutation detection and clonal tracking in cancer. *PLOS Comput. Biol.* **2020**, doi.org/10.1371/journal.pcbi.1007603.

17. Nakken, S.; Fournous, G.; Vodak, D.; Aasheim, L.B.; Myklebost, O.; Hovig, E. Personal Cancer Genome Reporter: variant interpretation report for precision oncology. *Bioinformatics* **2018**, *34*, 1778–1780, doi:10.1093/bioinformatics/btx817.

18. Sukhai, M.A.; Misyura, M.; Thomas, M.; Garg, S.; Zhang, T.; Stickle, N.; Virtanen, C.; Bedard, P.L.; Siu, L.L.; Smets, T., et al. Somatic Tumor Variant Filtration Strategies to Optimize Tumor-Only Molecular Profiling Using Targeted Next-Generation Sequencing Panels. *J. Mol. Diagn.* **2019**, *21*, 261–273, doi:10.1016/j.jmoldx.2018.09.008.
